# Understanding the Ligand Influence in the Multistep Reaction of Diazoalkanes with Palladium Complexes Leading to Carbene-Aryl Coupling

**DOI:** 10.1021/acs.organomet.4c00439

**Published:** 2025-01-10

**Authors:** Francisco Villalba, Ana C. Albéniz

**Affiliations:** IU CINQUIMA/Química Inorgánica, Universidad de Valladolid, Valladolid 47071, Spain

## Abstract

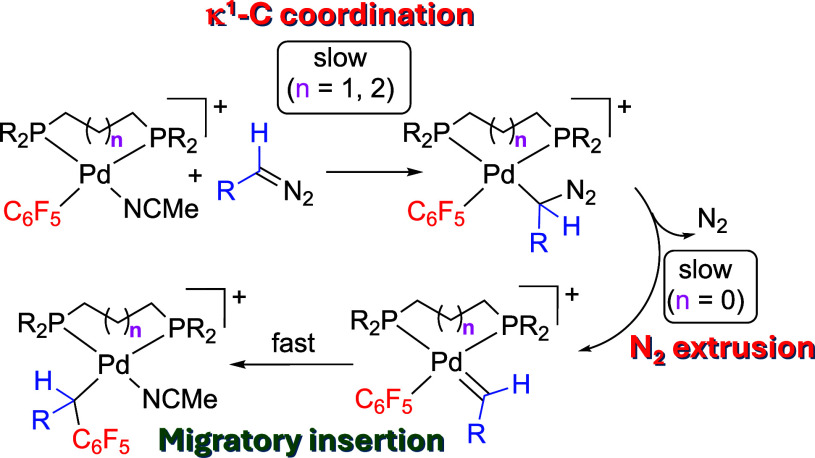

The reaction of diphosphino aryl complexes [Pd(C_6_F_5_)(L-L)(NCMe)](BF_4_) (L-L = dppe, dppp, dppb) with diazoalkanes N_2_CHR (*R* = −CH=CHPh, Ph) leads to η^3^-allyl or η^3^-benzyl palladium derivatives that are the organometallic products resulting from carbene-aryl coupling. The experimental trend shows that the reaction is favored for dppe > dppp > dppb. It involves several consecutive steps, i.e., diazoalkane coordination, nitrogen extrusion to give a Pd-carbene, and migratory insertion, which are experimentally inseparable, but they can be studied with the help of DFT calculations. The bulkiness and bite angle of the ligand exert a large influence in the relative rate of the steps involved in the reaction, and we have found that carbene formation by N_2_ extrusion is the step with the largest barrier for dppe. In contrast, the coordination of the diazoalkane is the most energy-demanding step for the larger dppp and dppb diphosphines. Thus, ligand substitution controls the rate, an important elemental step rarely considered in mechanistic studies of carbene cross coupling reactions. Since diazoalkanes are the most common carbene precursors, either directly or generated from hydrazones, the choice of ligand can be very important to facilitate the entrance of the carbene precursor in the catalytic cycle.

## Introduction

**Scheme 1:**
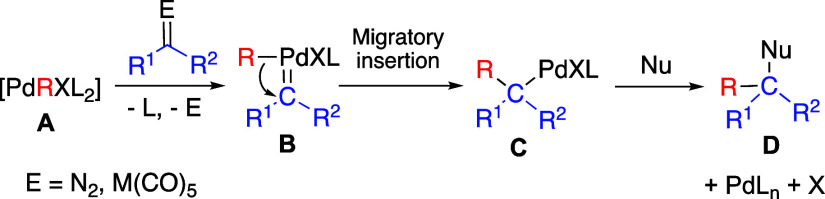
Intermediates in the Carbene-Hydrocarbyl Coupling from Carbene Precursors

Palladium-catalyzed cross coupling reactions that use carbene precursors as reagents are important processes in the C–C bond forming reaction toolbox. The carbene fragment is amenable to double functionalization and cascade reactions lead to the formation of two C–C or C-X bonds, which builds up molecular complexity in a fewer number of synthetic steps.^1,2^ The key step in these transformations is the 1,1-insertion (or migratory insertion) of a carbene fragment into a Pd–C bond in a metal carbene intermediate (**B**) formed by reaction of the carbene precursor and a palladium hydrocarbyl complex **A**. The detailed experimental study of this specific step (**B** to **C**, Scheme 1) is not easy because the preceding carbene complex is usually difficult to detect or isolate. Therefore, it is not possible to collect experimental data pertaining the rate of the migratory insertion step or the factors that favor it alone, since the observed outcome will be a combination of several consecutive steps. Very few isolated carbenes **B** have been shown to undergo a migratory insertion reaction and they are stabilized monoamino carbenes (R^1^ = NR_2_, R^2^ = alkyl, aryl, Scheme 1),^3^ and N-heterocyclic carbenes (NHCs).^4^ For carbenes with hydrocarbyl substituents or alkoxo groups the carbene is too unstable to be isolated and only the transformation **A** to **C** can be observed (Scheme 1).^3a,3c,5−7^

The most common carbene precursors used in Pd-catalyzed coupling processes are diazoalkanes N_2_R^1^R^2^, where R^1^, R^2^ = H, hydrocarbyl. They can be directly used as reagents or generated in situ via the decomposition of tosylhydrazones. Using these precursors the carbene intermediates **B** are too unstable to be detected but suitable substituents in the diazoalkane have allowed isolation of the alkyl complex right after the migratory insertion (**C**, Scheme 1). Thus, we have reported that using [Pd(C_6_F_5_)(dppe)(NCMe)](BF_4_) as precursor complex **A** it is possible to isolate derivatives of type **C** where the alkyl group is stabilized by coordination of a C–C double bond (η^3^-allylic complex) or a phenyl ring (η^3^-benzylic complex).^8^

With this system at hand, we decided to study the influence of the ligands in the formation of the migratory insertion product. In particular, we looked at the cis–trans stereochemistry of the complexes and the ligand bite angle for analogous chelating phosphines. The influence of the ligand bulk and bite angle on C–C bond formation reactions can be used to modulate a catalyst reactivity. For example, the increase of the reductive elimination rate upon introducing bulky ligands is known and has proved to be one of the most successful strategies to achieve efficient cross-coupling reactions in milder conditions or those of reluctant substrates (i.e fluorinated substates).^9,10^ 1,1-Migratory insertion reactions of carbon monoxide are also favored by bulky ligands with large bite angles and this has been shown for the insertion of CO into a Pd-alkyl bond. A series of well-defined methyl Pd(II)-complexes with bidentate phosphines, were examined by Brookhart et al. They observed that the kinetic barriers for the migratory insertion of CO into the Pd-methyl bond decrease with increasing the P–Pd–P bond angle of the complex and the steric bulk of the ligand.^11^ A large bite angle of the auxiliary ligand, brings the groups that have to couple close together (CO and Me in this case) and the barrier to reach the three membered transition state in the migratory insertion decreases.

Although the migratory insertion of CO and carbenes have many analogies (both are isolobal),^12^ no study of the influence of the ligands on the coupling of a carbene fragment and a Pd-R moiety has been reported. We have examined the reactivity of palladium aryl complexes with different monodentate and bidentate phosphine ligands and diazoalkanes, leading to C–C coupling complexes **C**. Because of the impossibility of isolating the relevant palladium carbene complex **B** and separating the migratory insertion step from the reaction of **A** with the diazo compound, both experimental and computational work is reported here. The combined experimental and computational data give information on the influence of the different steps in controlling the rate of the overall carbene-aryl coupling.

## Results and Discussion

### Preparation of the Palladium Phosphino Precursors

**Scheme 2:**
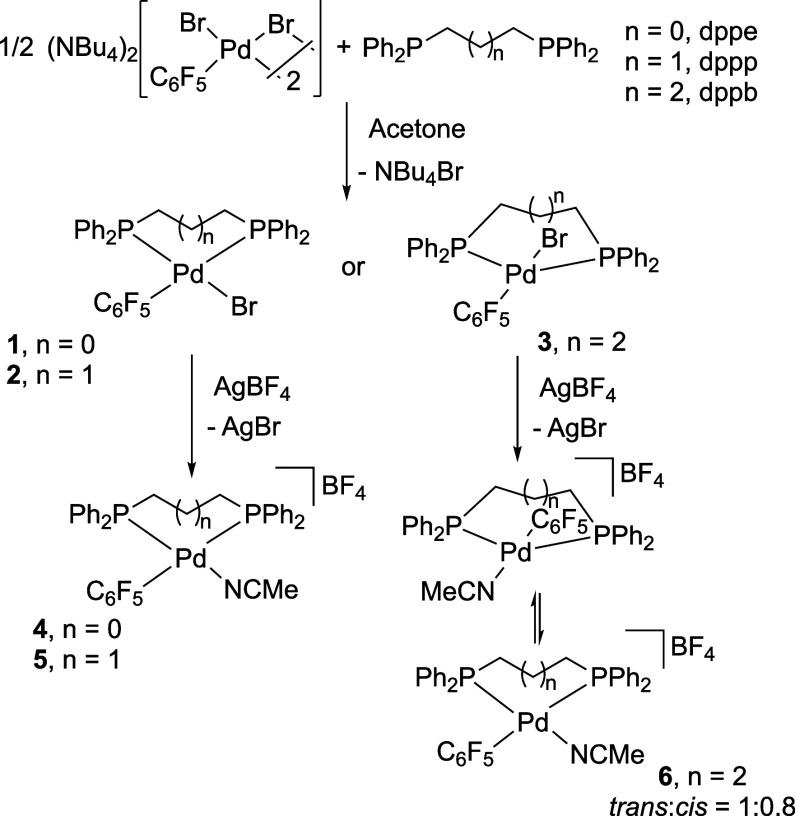
Synthesis of the Precursor Model Complexes

Complexes [PdBr(C_6_F_5_)(L-L)] (**1**-**3**), [Pd(C_6_F_5_)(L-L)(NCMe)](BF_4_) (**4**-**6**; L-L = dppe, dppp, dppb) (Scheme 2) and [Pd(C_6_F_5_)(NCMe)(PPh_3_)_2_](BF_4_) (**7**) were used as models to evaluate the ligand-dependent reactivity with diazoalkanes N_2_CH–CH=CHPh (**8**) and N_2_CHPh (**9**). These diazoalkanes have already proved to be suitable for stabilizing the expected alkyl intermediate after the migratory insertion step (**C**, Scheme1), by coordination of the unsaturated double bond or aryl group to the metal.^8^ The chosen phosphine ligands belong to the same family of diphosphines. Their electronic properties are very similar, but the tether between the phosphorus atoms and therefore the bite angle (P-M-P) is different in each phosphine. The use of pentafluorophenyl as a model aryl moiety is convenient since it allows the very informative follow up of the reactions by ^19^F NMR (see below), while showing analogous behavior to less fluorinated aryls in palladium-mediated C–C coupling processes.^13^ The syntheses of complexes **1**-**3** were carried out using the same dimeric precursor (NBu_4_)_2_[Pd(μ-Br)_2_Br_2_(C_6_F_5_)_2_] in the presence of the stoichiometric amount of the chelating ligand. Complex **1** has been reported before,^14^ and the molecular structure of the dppp derivative **2** was determined by X-ray diffraction. It shows a palladium square-planar geometry and a *cis* arrangement of the C_6_F_5_ and Br ligands (Figure S5, Supporting Information). The P–Pd–P and C–Pd–Br angles are 93.44° and 88.28° respectively, being in the expected range for other similar molecular structures of [PdAr(dppp)(X)] complexes reported in the literature.^15^ The *cis* arrangement is also present in solution as clearly shown by the appearance of two inequivalent ^31^P NMR resonances (see Experimental section). A different behavior was observed for dppb and the isolated complex **3** is a *trans*-species as shown by the appearance of only one ^31^P NMR resonance at 18.92 ppm. In chloroform solution at room temperature, **3** isomerizes to give a *trans*:*cis* mixture in a 0.8:1 mol ratio after 48 h (cf. Figures S14–S17, Supporting Information). When the isolated complex *trans*-**3** is treated with AgBF_4_ to remove the Br ligand in acetonitrile at room temperature, complex **6** was obtained as a mixture of isomers *trans*:*cis* = 1:0.8 mol ratio (Scheme 2). This was observed by ^19^F and ^31^P NMR (Figures S22, S23, Supporting Information). This behavior is only observed for the dppb ligand because of its inherent wide P–Pd–P angle in comparison to the dppe or dppp ligands. For the complexes bearing the latter ligands (**1** and **2**) their reaction with AgBF_4_ affords the corresponding *cis*-solvento acetonitrile derivatives **4** and **5** (Scheme 2). Complex *trans-*[Pd(C_6_F_5_)(NCMe)(PPh_3_)_2_](BF_4_) (**7**) was prepared in the same way from the known *trans-*[PdBr(C_6_F_5_)(PPh_3_)_2_] complex.^14^

The diphosphine 1,1-bis(diphenylphosphino)methane (dppm), with the smallest bite angle, was excluded from the study since the parent palladium complex of composition “PdBr(C_6_F_5_)dppm” was obtained as a mixture of the binuclear [Pd(μ-dppm)Br(C_6_F_5_)]_2_ (58%) with a bridging dppm and the monomeric [PdBr(C_6_F_5_)dppm] (42%). The percentages given were obtained from the crude reaction mixture by ^19^F and ^31^P NMR integration. This behavior of dppm has been observed before,^16^ and the presence of the bridging phosphine does not allow to evaluate the influence of the bite angle properly.

### Reactions of the Solvento Palladium Complexes with Diazo Compounds

**Scheme 3:**
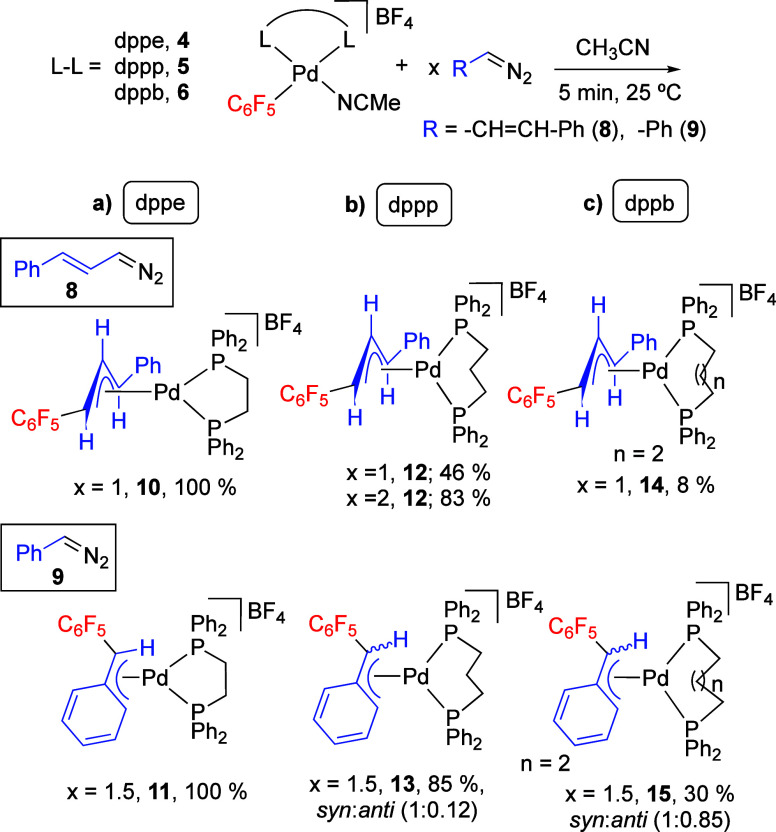
Complexes Formed upon Reaction of 4-6 with the Diazoalkanes

The solvento acetonitrile complexes readily react with diazoalkanes **8** and **9** giving organometallic η^3^-allylic complexes or η^3^-benzylic complexes respectively (Scheme 3). They are the result of the aryl-carbene coupling and correspond to the stabilized alkyl derivative **C** in Scheme 1. The reactions were carried out under the same conditions for all the complexes and the amounts of coupling products were determined by integration of the corresponding ^19^F signals in the NMR spectra of the reaction mixtures at a fixed reaction time (Scheme 3).

**Figure 1:**
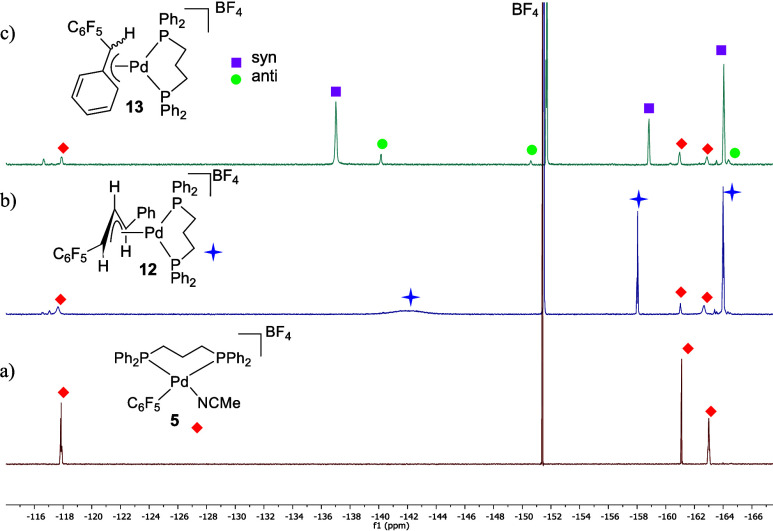
^19^F NMR spectra (470.17 MHz, CH_3_CN, (CD_3_)_2_SO capillary) of: a) complex **5**; b) the reaction of **5** with diazoalkane **8**, to give complex **12** (Pd:**8** = 1:2 mol ratio); c) the reaction of **5** with diazoalkane **9** to give **13** as a mixture of *syn* (depicted) and *anti* isomers.

**Figure 2:**
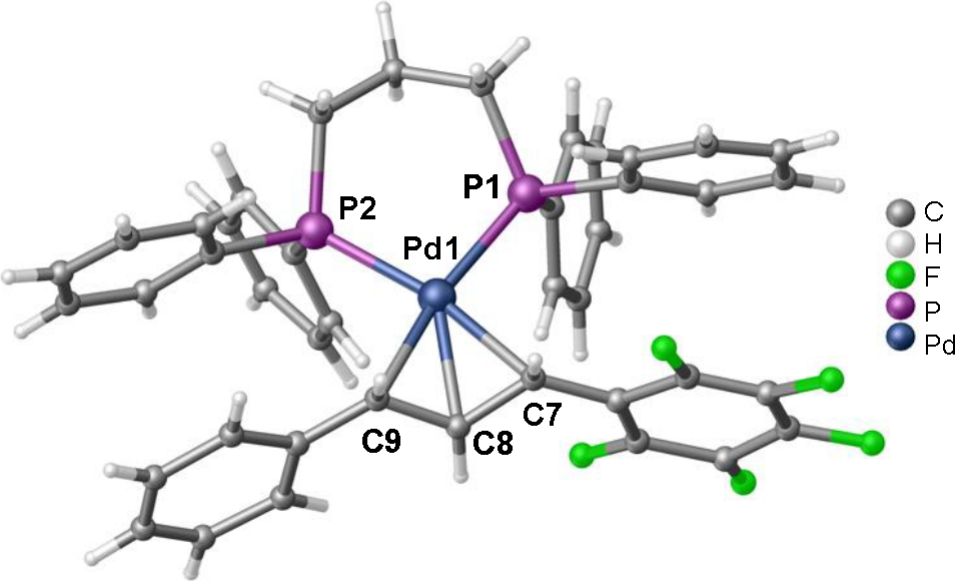
X-ray molecular structure of **12**. Solvent molecules **(**CHCl_3_**)** and the BF_4_^–^ anion are omitted for clarity. Selected bond lengths (Å) and angles (deg): Pd1–P2, 2.3035(17); Pd1–P1, 2.3057(16); Pd1–C9, 2.229(6); Pd1–C8, 2.196(6), Pd1–C7, 2.226(7); C7–C8, 1.406(10); C8–C9, 1.397(10).

We have reported the formation of complexes **10** and **11** by reaction the dppe derivative **4** with the diazoderivatives in full conversion after 5 min at room temperature (Scheme 3a) as well as their structural characterization.^8^ The characteristic ^19^F NMR signals of the starting solvento complex **4** (Pd–C_6_F_5_, F_ortho_ about −120 ppm) disappear and are replaced by the characteristic C–C_6_F_5_ resonances (F_ortho_ about −140 ppm) of the organometallic complexes obtained after the migratory insertion (Figure S1, Supporting Information). The reaction of the analogous dppp solvento complex **5** with an equimolar amount of diazoalkane **8** afforded 46% of the η^3^-allylic Pd complex **12**. An additional portion of diazoalkane was added to the same sample, and the reaction proceeded to reach 83% of **12** and a 17% of the starting **5** which remains unreacted (Scheme 3b). Characteristic signals for the migration of the C_6_F_5_ group to the carbene fragment were observed in the ^19^F NMR (F_ortho_ c.a −142 ppm, Figure 1, b). The F_ortho_ signals are broad showing a restricted rotation of the C–C_6_F_5_ bond at room temperature, presumably caused by the large bite angle and increased steric hindrance of the dppp ligand. The molecular structure of the η^3^-allyl complex **12** was determined by X-ray diffraction and it shows that both aryl-substituents of the η^3^-allylic fragment are in a *syn* arrangement (Figure 2).

The reaction of **5** with the diazoalkane **9** (Pd:**9** = 1:1.5 mol ratio) leads to the η^3^-benzylic palladium complex **13**, as a mixture of the *syn* pentafluorophenyl complex and a small amount of a tentatively assigned *anti*-C_6_F_5_ complex as collected in Scheme 3b and shown in Figure 1c.

As it was mentioned above the freshly prepared complex **6** is a mixture of *trans*:*cis* isomers (Scheme 2). This introduces a new factor that can distort the observed experimental results since the migratory insertion requires a *cis* arrangement of the carbene and hydrocarbyl fragments. The reaction of **6** with diazoalkane **8** afforded only 8% of the η^3^-allyl-palladium complex **14** (Scheme 3c). The remaining starting complex **6** is a mixture of the *trans*:*cis* isomers in a different ratio to that observed minutes before its *in situ* preparation (Figure S3, Supporting Information). This means that the *cis*-*trans* equilibrium, presumably slow, was not established at the beginning of the reaction. The analysis of the final reaction mixture indicates that the *cis*-**6** isomer is the major one, so the poor formation of the η^3^-allyl-palladium complex **14** is not governed by the lack of *cis*-**6**, although it can certainly be influenced by the lower concentration of this isomer in the starting mixture of complexes.

The reaction of **6** and the diazoalkane **9** leads to two new organometallic species (30% of the total amount of C_6_F_5_ in ^19^F NMR) which have been tentatively assigned to the *anti*:*syn* isomers of the η^3^-benzyl-organometallic product **15**, in an almost equimolar ratio (Figures S4, S39 and S40, Supporting Information). The *syn*-isomer shows characteristic chemical shifts in the ^19^F NMR analogous to the data for complex **11** (*syn*) that has been unequivocally characterized (F_ortho_ resonances at about −137 ppm (*syn*) vs −140 ppm (*anti*)).^8^ As can be seen in Scheme 3, the syn arrangement in the η^3^-benzylic complexes is preferred to the *anti* arrangement but the latter gains importance as the bulkiness of the ligands increase. This is confirmed by DFT calculations which show that the *syn* isomer is more stable for the dppe derivative by 1.62 kcal mol^–1^, but the energy difference is almost the same for both isomers in the case of the more sterically demanding dppp and dppb, in agreement with the experimental observations (Figure S41, Supporting Information).

*Trans-*[Pd(C_6_F_5_)(NCMe)(PPh_3_)_2_](BF_4_) (**7**) reacts with the diazoalkanes to give almost no aryl migration species (eq 1). When a mixture of **7** and the diazoderivative **8** was analyzed, we could not identify any organometallic product from the migratory insertion of a transient palladium carbene complex into the Pd–C_6_F_5_ bond and just small amounts of C_6_F_5_-containing organic products (5%) were detected by ^19^F NMR. Only decomposition products of the diazo compound were observed by ^1^H NMR: 5-Ph-1H-pyrazole, formed by cyclization of the diazoalkane **8**, and 1,6-diphenylhexa-1,3,5-triene, as result of the dimerization of the carbene fragment. Similar results were obtained in the reaction of **7** and diazoalkane **9** where no organometallic migratory insertion products could be detected by ^19^F NMR. Benzaldehyde, *cis*/*trans* stilbene and the azine Ph–CH = N–N = CH-Ph were detected by NMR as decomposition products of the diazoalkane **9**. For the monodentate phosphine precursor, the putative palladium carbene generated would be a transient *trans*-[Pd(C_6_F_5_)(PPh_3_)_2_(carbene)]^+^ and in this arrangement the migratory insertion cannot occur. Thus, the results obtained are consistent with a scenario where the isomerization process to afford a *cis* complex is slower than the decomposition of both the free diazoalkane and the metal carbene.
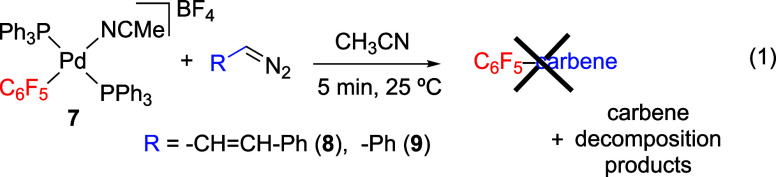
1

### DFT Calculations on the Carbene Formation and Migratory Insertion Steps

**Scheme 4:**
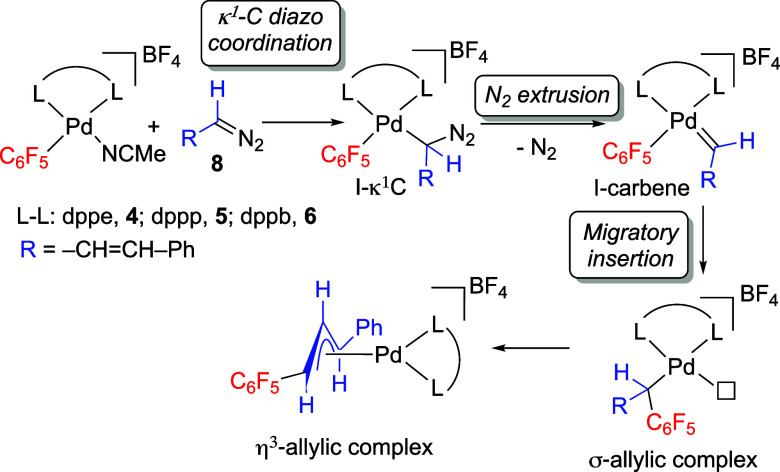
Reaction Pathway Leading to Aryl-Carbene Coupling Products

The nature of the auxiliary ligand and the different number of carbons in the backbone of the diphosphine ligands exert a relevant influence in the outcome of the reaction with diazoalkanes. For the chelating diphosphines the reactivity order that can be extracted from Scheme 3 roughly follows the trend: dppe > dppp > dppb. In a simplified way, the steps involved in the reactions of solvento acetonitrile Pd(II) complexes with diazoalkane **8** are depicted in Scheme 4.

**Figure 3:**
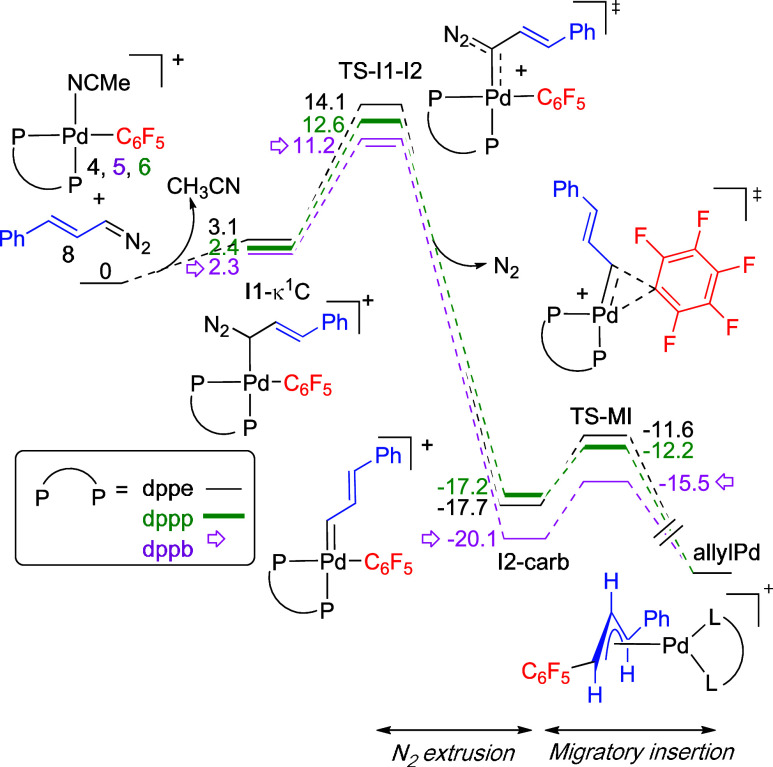
Gibbs energy profile for the N_2_ extrusion and migratory insertion steps (energies in kcal mol^–1^).

Only the η^3^-allylic products after migratory insertion were detected, so the coordination of the diazoalkane, the formation of the intermediate palladium carbene and the migratory insertion reaction cannot be experimentally studied separately. Attempts at detecting intermediate species at low temperature (−90 °C) were reported before for the dppe precursor with no success.^8^ For this reason, DFT calculations were employed to gain insight into the steps that are responsible for the differences observed. We modeled and compared the energy profiles for the reactions of dppe, dppp and dppb with diazoalkane **8** using the M06 functional and including solvation (MeCN) through the SMD implicit solvent method (see computational details in the Experimental part). First, we analyzed the nitrogen extrusion and migratory insertion steps. Figure 3 shows a general profile for the three phosphines and the energy values for the intermediates and transition states (for full specific energy profiles for each phosphine, see the Supporting Information, section 4).

The activation barriers for the migratory insertion step, i.e. Δ*G*_**TS-MI**_–Δ*G*_**I2-carb**_, follow the trend: dppe (6.1 kcal mol^–1^) > dppp (5 kcal mol^–1^) ≥ dppb (4.6 kcal mol^–1^). As the bite angle of the phosphine increases the barrier slightly decreases. This is more noticeable on going from dppe to dppp and it is the same trend observed for the migratory insertion reaction of CO.^11^ The angle C(C_6_F_5_)–Pd–C(carbene) in the carbene intermediate (**I2-carb**) is indeed smaller as the bite angle of the phosphine increases and this geometrical parameter is closer to the small angle required in the transition state (see Table S5, Supporting Information).

The barriers for nitrogen extrusion are higher than those for the migratory insertion step, as has also been found for a few other calculated systems.^17^ As shown in Figure 3, the ease of carbene formation (**I2-carb**) from the coordinated diazo compound (**I1-κ**^**1**^**-C**) for the three diphosphines follow the same trend found for the migratory insertion step, i.e. Δ*G*_**TS–I1-I2**_–Δ*G***_I1-κ_^1^_–C_**: dppe (11 kcal mol^–1^) > dppp (10.2 kcal mol^–1^) > dppb (8.9 kcal mol^–1^). These results do not fit with those observed experimentally. The efficiency in the formation of the migratory insertion products, i.e. dppe > dppp > dppb follows the opposite trend to that expected from the barriers in Figure 3. Therefore, we decided to explore the other step that is also involved in the reaction, i.e. the coordination of the diazoalkane to the palladium center to give intermediate **I1-κ^1^-C**.

### Diazoalkane Coordination

Diazoalkanes are ambidentate ligands that can coordinate to the metal using the terminal N (κ^1^-N) or the C (κ^1^-C) as donor atoms. Both intermediates were calculated for dppe and dppp, and they are close in energy although only the κ^1^-C coordinated diazoalkane evolves to the formation of the carbene complex (Figures S42–S43, Supporting Information).

**Figure 4:**
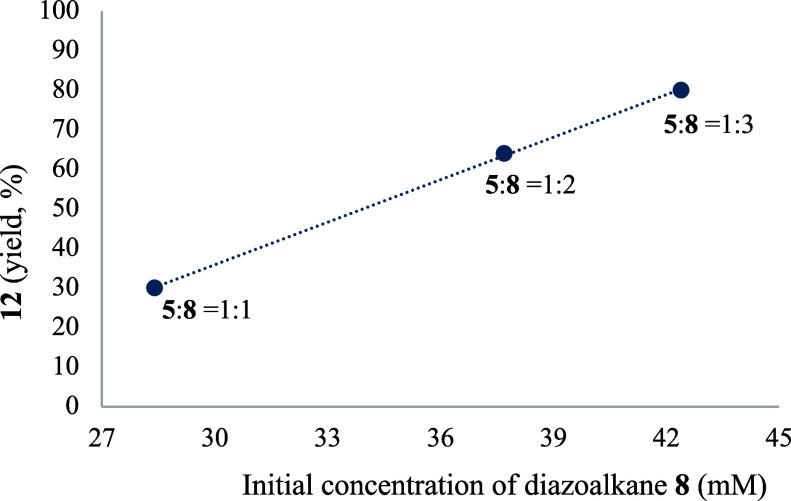
Amount of complex **12** formed by reaction of **5** ([**5**]_0_ = 28.4 mM) and different Pd:**8** mol ratios after 5 min at room temperature.

Both an associative substitution, where the transition state is a pentacoordinated trigonal-bipyramidal species, or a dissociative pathway, via a three-coordinated intermediate by dissociation of acetonitrile, could be possible (see the Supporting Information, section 4.3, for the dppe complexes). However, the reaction of the dppp complex **5** with diazoalkane **8** is informative and favors one of these pathways. Scheme 3 shows that the formation of the migratory insertion product **12** was not complete when the reaction was carried out with a Pd:**8** = 1:1 mol ratio for 5 min. However, the addition of another portion of diazoalkane **8** to the same sample increased the amount of **12**. To test the diazoalkane concentration dependence, three separate reactions with the same initial concentration of the solvento acetonitrile complex **5** ([**5**] = 28.4 mM) and different Pd:**8** mol ratios were carried out. Figure 4 shows the formation of **12** (%) when diazoalkane **8** was added in a Pd:**8** = 1, 2, and 3 mol ratio after 5 min at room temperature. The values depicted in the plot show an increase of the reaction rate upon diazoalkane concentration and this points to an associative pathway, which was the one modeled for the system.

**Figure 5:**
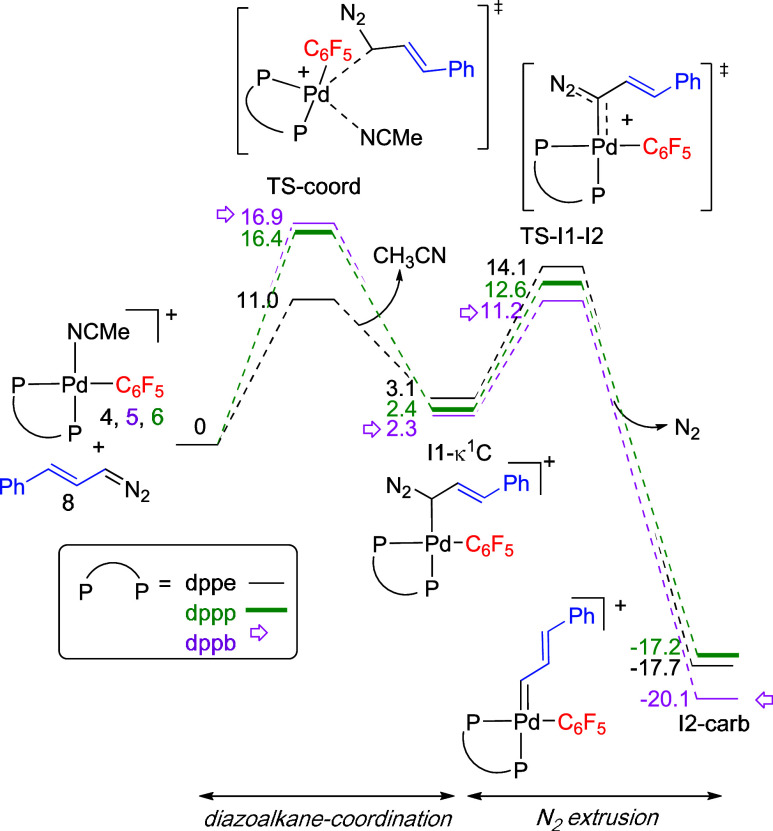
Gibbs energy profile for the diazoalkane coordination and N_2_ extrusion steps (energies in kcal mol^–1^).

Figure 5 shows the energy profiles for the diazoalkane coordination to palladium as well as the N_2_-extrusion step to generate the palladium carbene. The coordination of the diazoalkane becomes more energy demanding on going from dppe to the bulkier dppp and dppb. This effect is important and in fact, Figure 5 shows that the rate-controlling step for the overall carbene-aryl coupling in the dppe complex is the N_2_ extrusion to form the palladium carbene (energy barrier 14 kcal mol^–1^). In contrast, the process for the bulkier phosphines is controlled by the coordination of the diazoalkane to give intermediate **I1-κ**^**1**^**-C** (energy barrier around 16–17 kcal mol^–1^). According to this, the expected reactivity trend wouldbe dppe (N_2_-extrussion) > dppp ≥ dppb (diazoalkane coordination), consistent with the experimental trend.

These results show the importance of the diazoalkane coordination and how this step can be easily affected by the steric bulk of the ligand, therefore controlling the overall reaction rate. Many catalytic cross-coupling reactions use hydrazones that slowly decompose to diazoalkanes providing a usually low concentration of the latter in the reaction medium. Under these conditions, the choice of ligands and the study of the ligand substitution step can be crucial to avoid a too demanding diazoalkane coordination and to ensure an efficient catalysis. For example, Dingwall et al. carried out an experimental mechanistic study on the Pd-catalyzed cross coupling of a diazoalkane with benzyl bromide and determined that the palladium carbene formation, i.e. the overall reaction of the diazoalkane with a palladium benzyl complex, is turnover limiting in a Pd-phosphine system. The diazo coordination could be responsible for this, but the ligand substitution step was not calculated separately.^18^

## Conclusions

The reaction of diazoalkanes and palladium-hydrocarbyl complexes is involved in many catalytic processes that lead to carbene-hydrocarbyl cross-coupling products. The reaction is a multistep process that involves several elemental reactions (diazoalkane coordination, nitrogen extrusion to give a Pd-carbene, and migratory insertion) whose rates can be influenced by the auxiliary ligands.

The experimental trend for the ease of formation of carbene-aryl coupling products in the reaction of the solvento complexes [Pd(C_6_F_5_)(P–P)(NCMe)]BF_4_ with diazoalkanes for different ancillary diphosphine ligands is P–P = dppe > dppp > dppb. Since the electronic features of these phosphines are similar, the observed differences in the formation of the migratory insertion organometallic complexes can be attributed primarily to the ligand backbone. DFT calculations show that the trend in energy barriers for nitrogen extrusion and migratory insertion do not mirror the experimental outcome, since the bulkier phosphines show lower reaction barriers. The experimental differences can be explained considering the rates of coordination of the diazoalkane to palladium. We have found that the coordination of the diazoalkane **8** to the palladium complex bearing a dppe ligand has a low activation energy and the nitrogen extrusion in a κ^1^-C coordinated diazoalkane is the rate controlling step of the reaction. In contrast, the dppp and dppb complexes show a higher activation barrier for the coordination of the diazoalkane, via an associative pathway. Therefore, the coordination of the diazoalkane is controlling the overall reaction rate for large bite angle phosphines.

The coordination of the diazoalkane is often overlooked in mechanistic studies on carbene-hydrocarbyl couplings catalyzed by palladium complexes, which usually concentrate on the carbene formation by N_2_ extrusion and migratory insertion to explain either rate or selectivity, or both. The importance of ligand substitution reactions in catalysis cannot be underestimated and the results here show that this is also the case in carbene-hydrocarbyl couplings.

The reaction of *trans-*[Pd(C_6_F_5_)(NCMe)(PPh_3_)_2_]BF_4_ with diazoalkanes does not lead to carbene-aryl coupling. This fact clearly evidence that the *trans* arrangement hampers the migratory insertion process and that the required isomerization to a *cis* complex can be slower than the decomposition pathways of the diazoalkane and the *trans*-palladium carbene species.

## Experimental Section

### General Methods

^1^H, ^13^C{^1^H} ^31^P{^1^H} and ^19^F NMR spectra were recorded on an Agilent MR-500 spectrometer at the *Laboratorio de Técnicas Instrumentales* (LTI) of the UVa. Chemical shifts (in δ units, ppm) were referenced to SiMe_4_ (^1^H and ^13^C), CFCl_3_ (^19^F) and H_3_PO_4_ (85%, ^31^P). The spectral data were recorded at 298 K unless otherwise noted. Homonuclear (^1^H–COSY) and heteronuclear (^1^H–^13^C HSQC and HMBC) NMR experiments were used to help with the signal assignments. Elemental analyses were carried out in a Carlo Erba 1108 microanalyzer (at the Vigo University, Spain). All reactions were conducted under a N_2_ atmosphere. Solvents were dried using a solvent purification system SPS PS-MD-5 (ether, hexane, THF and CH_2_Cl_2_) or distilled from appropriate drying agents under nitrogen prior to use and stored over 3 or 4 Å molecular sieves (acetonitrile and acetonitrile-d_3_). All commercial reagents and solvents were used as received unless otherwise indicated. Complexes (NBu_4_)_2_[Pd(μ-Br)_2_Br_2_(C_6_F_5_)_2_],^14^ [PdBr(C_6_F_5_)(dppe)] (**1**),^14^ [Pd(C_6_F_5_)(dppe)(NCMe]BF_4_ (**4**),^8^ and [PdBr(C_6_F_5_)(PPh_3_)_2_]^14^ were prepared according to the literature methods. Complexes **10** and **11** have been reported and characterized before.^8^ The syntheses of the diazo compounds were carried out according to the literature methods.^19^ The diazoalkanes were prepared and kept as dichloromethane solutions for no longer than 10 days under a nitrogen atmosphere at −28 °C in the dark. The concentrations of these solutions were determined by ^1^H NMR using CF_3_CH_2_I as internal standard.

### Synthesis of [PdBr(C_6_F_5_)(dppp)] (2)

1,3-Bis(diphenylphosphino)propane (dppp) (110.87 mg, 0.268 mmol) was added to a solution of (NBu_4_)_2_[Pd(μ-Br)_2_Br_2_(C_6_F_5_)_2_] (176.5 mg, 0.130 mmol) in acetone (30 mL). The mixture was stirred at room temperature for 1 h. During this time the orange solution became pale-yellow. The solvent was evaporated to dryness and the yellow oil was triturated with cold EtOH until the formation of a pale-yellow solid that was filtered, washed with cold EtOH and air-dried. Yield: 165 mg (83%). Crystals suitable for X-ray analyses were obtained by slow evaporation of a solution of **2** in CHCl_3_. ^1^H NMR (499.73 MHz, δ, CDCl_3_): 7.78–7.73 (m, 4H, H^arom^), 7.50–7.42 (m, 6H, H^arom^), 7.41–7.33 (m, 6H, H^arom^), 7.17 (td, J = 5.5 Hz, 2.4 Hz, 4H, H^arom^), 2.66 (m, 2H, CH_2_), 2.33 (m, 2H, CH_2_), 2.03 (m, 2H, CH_2_). ^13^C{^1^H} NMR (125.67 MHz, δ, CDCl_3_): 138.3 (d, J_C–P_ = 11.2 Hz, C^arom^), 133.4 (d, J_C–P_ = 10.6 Hz, C^arom^), 132.7 (d, J_C–P_ = 11.0 Hz, C^arom^), 131.1 (d, J_C–P_ = 2.6 Hz, C^para^), 130.8 (d, J_C–P_ = 2.5 Hz, C^para^), 130.4 (d, J^1^_C–P_ = 45.7 Hz, C^ipso^), 130.1 (d, J^1^_C–P_ = 54.7 Hz, C^ipso^), 128.7 (d, J_C–P_ = 10.3 Hz, C^arom^), 25.8 (dd, J = 29.2, 7.3 Hz, CH_2_), 25.4 (dd, J = 25.2, 7.4 Hz, CH_2_), 18.9 (s, CH_2_).* ^19^F NMR (470.17 MHz, δ, CDCl_3_): −116.92 (m, 2F, F_ortho_), −161.77 (t, *J* = 20.1 Hz, 1F, F_para_), −162.85 (m, 2F, F_meta_). ^31^P{^1^H} NMR (202.31 MHz, δ, CDCl_3_): 13.60 (dt, J = 42.4 Hz, 6.4 Hz, 1P), −5.82 (m, 1P). Anal. Calcd for C_33_H_26_BrF_5_P_2_Pd: C, 51.76%; H, 3.42%. Found: C, 51.60%; H, 3.26%. *The ^13^C signals for the C_6_F_5_ group, heavily coupled to ^19^F, could not be observed.

### Synthesis of *trans*-[PdBr(C_6_F_5_)(dppb)] (*trans*-3)

1,4-Bis(diphenylphosphino)butane (dppb) (111.55 mg, 0.256 mmol) was added to a solution of (NBu_4_)_2_[Pd(μ-Br)_2_Br_2_(C_6_F_5_)_2_] (173.0 mg, 0.128 mmol) in acetone (30 mL). The mixture was stirred at room temperature for 1 h. During this time the orange solution became pale-yellow. The solvent was evaporated to dryness and the yellow oil was triturated with cold EtOH until the formation of a pale-yellow solid that was filtered, washed with cold EtOH and air-dried. Yield: 175 mg (88%). ^1^H NMR (499.72 MHz, δ, CDCl_3_): 7.43 (m, 9H, H^arom^), 7.27 (t, J = 7.2 Hz, 4H, H^arom^), 7.21 (m, 7H, H^arom^), 2.63 (m, 4H, CH_2_), 2.01 (m, 4H, CH_2_). ^13^C{^1^H} NMR (125.67 MHz, δ, CDCl_3_): 132.9 (br, C^arom^), 131.4 (d, J^1^_C–P_ = 47.1 Hz, C^arom^), 130.2 (br, C^arom^), 128.1 (br, C^arom^), 27.5 (m, 4C, CH_2_). ^19^F NMR (470.17 MHz, δ, CDCl_3_): −116.19 (m, 2F, F_ortho_), −161.59 (t, *J* = 19.7 Hz, 1F, F_para_), −162.22 (m, 2F, F_meta_). ^31^P{^1^H} NMR (202.31 MHz, δ, CDCl_3_): 18.92 (s, 2P). Anal. Calcd for C_34_H_28_BrF_5_P_2_Pd: C, 52.36%; H, 3.62%. Found: C, 52.56%; H, 3.68%. When a solution of complex *trans*-**3** was kept at room temperature for 48 h in CDCl_3_ a mixture of isomers (*trans*:*cis* = 0.8:1) was formed. *cis*-**3**: ^19^F NMR (470.17 MHz, δ, CDCl_3_): −117.15 (m, 2F, F_ortho_), −161.99 (t, *J* = 19.9 Hz, 1F, F_para_), −162.83 (m, 2F, F_meta_). ^31^P{^1^H} NMR (202.31 MHz, δ, CDCl_3_): 40.72 (d, J = 32.5 Hz, 1P), −1.40 (m, 1P).

### Characterization of [Pd(C_6_F_5_)(dppp)(NCMe)](BF_4_) (5)

[PdBr(C_6_F_5_)(dppp)] (13.4 mg, 0.017 mmol) and AgBF_4_ (3.4 mg, 0.017 mmol) were mixed in dry MeCN (0.6 mL) and stirred for 15 min at room temperature under nitrogen. The suspension was filtered through Kieselguhr to remove the AgBr and the resulting colorless solution was characterized by NMR. Upon isolation attempts some reorganization of the aryl groups occurs by transmetalation and the solids obtained were inevitably contaminated by small amounts of “Pd(C_6_F_5_)_2_” derivatives. Therefore, the complexes were usually synthesized in situ and used in solution.

^1^H NMR (499.73 MHz, δ, CH_3_CN/(CD_3_)_2_SO capillary): 7.94–7.83 (m, 10H, H^arom^), 7.71–7.64 (m, 6H, H^arom^), 7.52 (m, 4H, H^arom^), 3.18 (m, 2H, CH_2_), 2.98 (m, 2H, C’H_2_).* ^19^F NMR (470.17 MHz, δ, CH_3_CN,(CD_3_)_2_SO capillary): −117.87 (m 2F, F_ortho_), −151.42 (BF_4_), −161.10 (t, J = 19.2 Hz, 1F, F_para_), −162.99 (m, 2F, F_meta_). ^31^P{^1^H} NMR (202.31 MHz, δ, CH_3_CN, (CD_3_)_2_SO capillary): 16.50 (dt, J = 39.7, 7.2 Hz, 1P), −4.39 (m, 1P). * One CH_2_ from dppp is overlapped with the NCCH_3_ signal.

### Characterization of [Pd(C_6_F_5_)(dppb)(NCMe)](BF_4_) (6)

[PdBr(C_6_F_5_)(dppb)] (52.0 mg, 0.066 mmol) and AgBF_4_ (13.0 mg, 0.066 mmol) were mixed in dry MeCN (0.6 mL) and stirred for 15 min at room temperature under nitrogen. The suspension was filtered through Kieselguhr to remove the AgBr and the resulting colorless solution was characterized by NMR. The resulting complex is a mixture of *trans:cis* = 1:0.8 isomers. ^1^H NMR (499.73 MHz, δ, CH_3_CN/(CD_3_)_2_SO capillary; *cis*-**6 +***trans*-**6**): 7.92 (m, 1H, H^arom^), 7.85 (m, 3H, H^arom^), 7.80 (m, 2H, H^arom^), 7.77–7.69 (m, 10H, H^arom^), 7.66 (m, 2H, H^arom^), 7.57 (td = J = 7.8, 2.9 Hz, 2H, H^arom^). The CH_2_ signals of the dppb ligand are overlapped with the NCCH_3_ signal. *cis*-**6**: ^19^F NMR (470.17 MHz, δ, CH_3_CN,(CD_3_)_2_SO capillary): −118.58 (m 2F, F_ortho_), −151.11 (BF_4_), −161.04 (t, J = 19.2 Hz, 1F, F_para_), −162.78 (m, 2F, F_meta_). ^31^P{^1^H} NMR (202.31, MHz, δ, CH_3_CN,(CD_3_)_2_SO capillary): 40.60 (dt, J = 30.8, 7.2 Hz, 1P), 7.26 (m, 1P). *trans*-**6**: ^19^F NMR (470.17 MHz, δ, CH_3_CN,(CD_3_)_2_SO capillary): −116.85 (m 2F, F_ortho_), −151.11 (BF_4_), −160.18 (t, J = 19.6 Hz, 1F, F_para_), −161.98 (m, 2F, F_meta_). ^31^P{^1^H} NMR (202.31, MHz, δ, CH_3_CN,(CD_3_)_2_SO capillary): 17.60 (s).

### Synthesis of [Pd(C_6_F_5_)(NCMe)(PPh_3_)_2_](BF_4_) (7)

Equimolar amounts of [PdBr(C_6_F_5_)(PPh_3_)_2_] (184.3 mg, 0.210 mmol) and AgBF_4_ (41 mg, 0.210 mmol) were mixed in dried CH_3_CN (10 mL) and stirred for 15 min at room temperature under nitrogen. The suspension was filtered through Kieselguhr and the filtrate was evaporated to dryness. The resulting yellow oil was triturated with *n*-hexane until the formation of a pale-yellow solid that was filtered, washed with *n*-hexane and air-dried. Yield: 118 mg, (60%). ^1^H NMR (499.73 MHz, δ, CD_3_CN): 7.62 (m, 6H, H_para_ PPh_3_) 7.60–7.50 (m, 24H, H_meta,ortho_ PPh_3_). ^13^C{^1^H} NMR (125.67 MHz, δ, CD_3_CN): 144.3 (m, ^1^J_C–F_ = 230.5 Hz, C_ortho_, C_6_F_5_), 138.2 (m, ^1^J_C–F_ = 250 Hz, C_para_, C_6_F_5_), 136.3 (m, ^1^J_C–F_ = 248 Hz, C_meta,_ C_6_F_5_), 133.7 (t, J_C–P_ = 6.5 Hz, C_ortho_ PPh_3_), 131.9 (C_para_ PPh_3_), 129.2 (t, J_C–P_ = 5.3 Hz, C_meta_ PPh_3_), 127.5 (t, J_C–P_ = 25.5 Hz, C_ipso_ PPh_3_). ^19^F NMR (470.17 MHz, δ, CD_3_CN): −118.40 (m, 2F, F_ortho_), −151.70 (BF_4_), −161.63 (tt, J = 19.0, 2.4 Hz, 1F, F_para_), −162.34 (m, 2F, F_meta_). ^31^P{^1^H} NMR (202.29, MHz, δ, CD_3_CN): 23.12 (td, J = 6.8, 2.2 Hz). Anal. Calcd for C_44_H_33_BF_9_NP_2_Pd: C, 57.08%; H, 3.59%; N, 1.51%. Found: C, 56.68%; H, 3.44%; N, 1.40%. *The ^13^C signals for the C_meta_ and C_ipso_ (C_6_F_5_ group) could not be observed.

### Characterization of [Pd(dppp)(η^3^-Ph–CH–CH–CH-C_6_F_5_)](BF_4_) (12)

[Pd(Br)(C_6_F_5_)(dppp)] (13.4 mg, 0.017 mmol) and AgBF_4_ (3.4 mg, 0.017 mmol) were mixed in dry MeCN (0.6 mL) and stirred for 15 min at room temperature under nitrogen. The suspension was filtered through Kieselguhr to remove the AgBr. Addition of a dichloromethane solution of the diazo compound N_2_CH–CH=CHPh (2-fold molar amount in two portions, 87 μL, 0.4 M, total of 0.046 mmol) afforded an intense yellow solution, which was stirred at room temperature for 5 min. Then, the solution was characterized by NMR. The crude yield was determined by integration of the ^19^F NMR signals in the mixture, (83%). Crystals suitable for X-ray analyses were obtained by slow diffusion of *n*-hexane layered onto a solution of the complex **12** in CHCl_3_ at −28 °C. ^1^H NMR (499.73 MHz, δ, CH_3_CN/(CD_3_)_2_SO capillary): 7.72–7.36 (m, 25H, H^arom^), 6.86 (t, J = 12.7 Hz, 1H, H^allyl^), 5.36 (t, J = 11.2 Hz, 1H, H^allyl^), 4.87 (t, J = 11.2 Hz, 1H, H^allyl^).* ^19^F NMR (470.17 MHz, δ, CH_3_CN, (CD_3_)_2_SO capillary): −141.99 (br, 2F, F_ortho_), −151.53 (BF_4_), −158.04 (t, J = 20.5 Hz, 1F, F_para_), −164.00 (m, 2F, F_meta_). ^31^P{^1^H} NMR (202.31, MHz, δ, CH_3_CN, (CD_3_)_2_SO capillary): AB system. ν_A_: 8.50 (d, J = 83.2 Hz, 1P), ν_B_: 7.06 (d, J = 83.2 Hz, 1P). * The CH_2_ signals of the dppp ligand are overlapped with the NCCH_3_ signal.

The analogous reactions for the dppb and PPh_3_ derivatives were carried out in the same way using an equimolar amount of the diazocompound. The formation of [Pd(dppb)(η^3^-Ph–CH–CH–CH-C_6_F_5_)](BF_4_) (**14**) was observed in 8% yield. ^1^H NMR (499.73 MHz, δ, CH_3_CN/(CD_3_)_2_SO capillary): 6.59 (t, J = 12.6 Hz, 1H, H^allyl^), 5.52 (t, J = 11.5 Hz, 1H, H^allyl^), 4.91 (t, J = 11.5 Hz, 1H, H^allyl^).* ^19^F NMR (470.17 MHz, δ, CH_3_CN, (CD_3_)_2_SO capillary): −140.31 (br, 1F, F_ortho_), −143.12 (br, 1F, F_ortho_), −151.64 (BF_4_). The F_para_ and F_meta_ as well as the ^31^P NMR signals have not been assigned due to the very low concentration of η^3^-allyl-complex in the reaction medium.

### Characterization of [Pd(dppp)(η^3^-Ph–CH-C_6_F_5_)](BF_4_) (13)

[Pd(Br)(C_6_F_5_)(dppp)] (17.7 mg, 0.023 mmol) and AgBF_4_ (4.5 mg, 0.023 mmol) were mixed in dry MeCN (0.6 mL) and stirred for 15 min at room temperature under nitrogen. The suspension was filtered through Kieselguhr to remove the AgBr. The addition of a dichloromethane solution of the diazo compound N_2_CHPh (0.0345 mmol, 128 μL, 0.27 M) afforded an intense yellow solution, which was stirred at room temperature for 5 min. Then, the solution was characterized by NMR. Two isomers were observed, *syn*:*anti* = 89:11, The crude yield was determined by integration of the ^19^F NMR signals in the crude mixture (85%). *syn*-**13**: ^1^H NMR (499.73 MHz, δ, CH_3_CN/(CD_3_)_2_SO capillary): 6.97 (m, 2H, H^2^, H^6^), 4.28 (d, J_H–P_ = 4.28 Hz, 1H, H^α^).* ^19^F NMR (470.17 MHz, δ, CH_3_CN, (CD_3_)_2_SO capillary): −137.02 (m 2F, F_ortho_), −151.72 (BF_4_), −158.84 (m, 1F, F_para_), −164.04 (m, 2F, F_meta_). ^31^P{^1^H} NMR (202.31, MHz, δ, CH_3_CN, (CD_3_)_2_SO capillary): 17.29 (dt, J = 81.2, 8.2 Hz, 1P), 4.94 (d, J = 81.2 Hz, 1P). *anti*-**13**: ^19^F NMR (470.17 MHz, δ, CH_3_CN, (CD_3_)_2_SO capillary): −140.16 (m 2F, F_ortho_), −150.59 (m, 1F, F_para_), −151.72 (BF_4_), −162.87 (m, 2F, F_meta_). * The remaining signals could not be assigned.

The reaction for the dppb and PPh_3_ derivatives were carried out in the same way. Complex **15** (dppb) was observed in 30% crude yield. *syn*-**15**: ^1^H NMR (499.73 MHz, δ, CH_3_CN/(CD_3_)_2_SO capillary): 6.96 (m, 2H, H^2^, H^6^), 4.17 (d, J = 11.9 Hz, 1H, H^α^).* ^19^F NMR (470.17 MHz, δ, CH_3_CN,(CD_3_)_2_SO capillary): −137.02 (m 2F, F_ortho_), −151.66 (BF_4_), −158.02 (m, 1F, F_para_), −163.94 (m, 2F, F_meta_). ^31^P{^1^H} NMR (202.31, MHz, δ, CH_3_CN,(CD_3_)_2_SO capillary): 36.55 (dt, J = 64.0 Hz, 1P), 11.31 (d, J = 64.0 Hz, 1P). *anti*-**15**: ^1^H NMR (499.73 MHz, δ, CH_3_CN/(CD_3_)_2_SO capillary): 6.64 (m, 2H, H^2^, H^6^), 4.72 (m, 1H, H^α^).* ^19^F NMR (470.17 MHz, δ, CH_3_CN,(CD_3_)_2_SO capillary): −140.02 (m 2F, F_ortho_), −151.14 (m, 1F, F_para_), −151.66 (BF_4_), −160.67 (m, 2F, F_meta_). ^31^P{^1^H} NMR (202.31, MHz, δ, CH_3_CN,(CD_3_)_2_SO capillary): 28.35 (d, J = 44.9 Hz, 1P), 14.95 (d, J = 44.9 Hz, 1P). *The remaining signals could not be assigned.

### Experiments for the Formation of Complex 12 at Different Diazoalkane Concentrations

[Pd(C_6_F_5_)(dppp)(NCMe)]BF_4_ (0.028 mmol) and 0.5 mL of dry CH_3_CN ([Pd]_0_ = 56 mM) were added into an NMR tube along with a sealed glass capillary filled with (CD_3_)_2_SO as NMR lock signal under a nitrogen atmosphere. Addition of a dichloromethane solution of the diazo compound N_2_CH–CH=CHPh (**8**) (Pd:**8** = 1:1, 1:2 and 1:3 mol ratio for each of the three experiments) afforded an intense yellow solution, which was stirred at room temperature for 5 min. Then, the solution was checked by ^19^F NMR. The crude yield of **12** was determined by integration of the ^19^F NMR signals in the mixture: Pd:**8** = 1:1; 30%. Pd:**8** = 1:2; 64%. Pd:**8** = 1:3; 80%. See Figure 4.

### Computational Methods

All calculations were performed using the DFT approach with the meta-hybrid GGA M06 functional,^20,21^ using Gaussian09 as program package.^22^ The selected basis set was 6-31+G(d) for C, N, F and H,^23,24^ and LANL2TZ(f) for Pd^25,26^ (Basis set I). Solvation was introduced in all the optimizations, frequency calculations and potential energy refinement through the SMD model, where we applied the experimental solvent, acetonitrile (ε = 37.5, at 25 °C). All geometry optimizations were carried out in solution with no symmetry restrictions. Free energy corrections were calculated at 298.15 K and 10^5^ Pa pressure, including zero-point energy corrections (ZPE), and the energies were converted to 1 M standard state in solution (adding/subtracting 1.89 kcal/mol for nonunimolecular processes). Vibrational frequency calculations were performed to establish the stationary points were minima (without imaginary frequencies) or transition states (with one imaginary frequency). Connectivity of the transition state structures were confirmed by relaxing the transition state geometry toward both the reactant and the product. Final potential energies were refined by performing additional single-point energy calculations (also in solution), Pd was still described with LANL2TZ(f) basis set, and the remaining atoms were treated with 6- 311++G(d,p) basis set (Basis set II). All reported energies in the manuscript correspond to Gibbs energies in solution, obtained from potential energies (including solvation) with basis set II plus Gibbs energy corrections with basis set I and are given in kcal mol^–1^.

## Supporting Information Available

Additional experimental data, selected spectra, computational data including calculated potential energies (PDF)
Coordinates for the calculated structures (XYZ)

## Supplementary Material

om4c00439_si_001.pdf

om4c00439_si_002.xyz
