# Supplementary material for: Understanding the Ligand Influence in the Multistep Reaction of Diazoalkanes with Palladium Complexes Leading to Carbene-Aryl Coupling
Source: Organometallics. 2025 Jan 10;44(2):394–402. doi: 10.1021/acs.organomet.4c00439 (PMC11776105; doi:10.1021/acs.organomet.4c00439)
Supplement: Supplementary file 1 — om4c00439_si_001.pdf [file om4c00439_si_001.pdf]

Supporting Information

Understanding the Ligand Influence in the Multistep Reaction of Diazoalkanes with Palladium Complexes Leading to Carbene-Aryl Coupling

Francisco Villalba and Ana C. Albéniz*
IU CINQUIMA/Química Inorgánica. Universidad de Valladolid. 47071 Valladolid (Spain)]
e-mail: albeniz@uva.es

Table of contents

1. Additional figures with experimental details.
2. Data for X-Ray molecular structure determinations
3. Selected NMR spectra
4. Computational details
    4.1 Selected figures and geometrical parameters
    4.2 Gibbs energy profiles
    4.3 Probing a dissociative diazoalkane substitution for complex **4** (dppe)
    4.4 Calculated energies
    4.5 IRC for selected transition states
5. References

1. Additional figures with experimental details

Reactions of the solvento aryl palladium complexes with diazoalkanes.

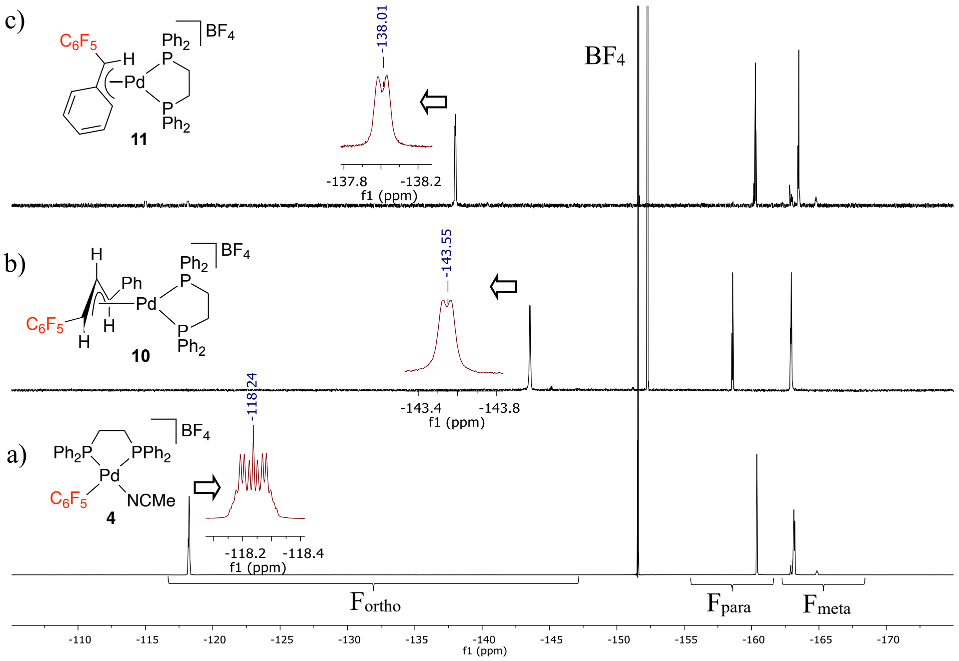

Figure S1. ^{19}F NMR spectra (470.17 MHz, CH_3CN, $(CD_3)_2SO$ capillary) of: a) complex **4**; b) the reaction of **4** with diazoalkane $N_2CH–CH=CHPh$ (**8**) to give complex **10**; c) the reaction of **4** with diazoalkane N_2CHPh (**9**) to give **11** (see text, Scheme 3a).

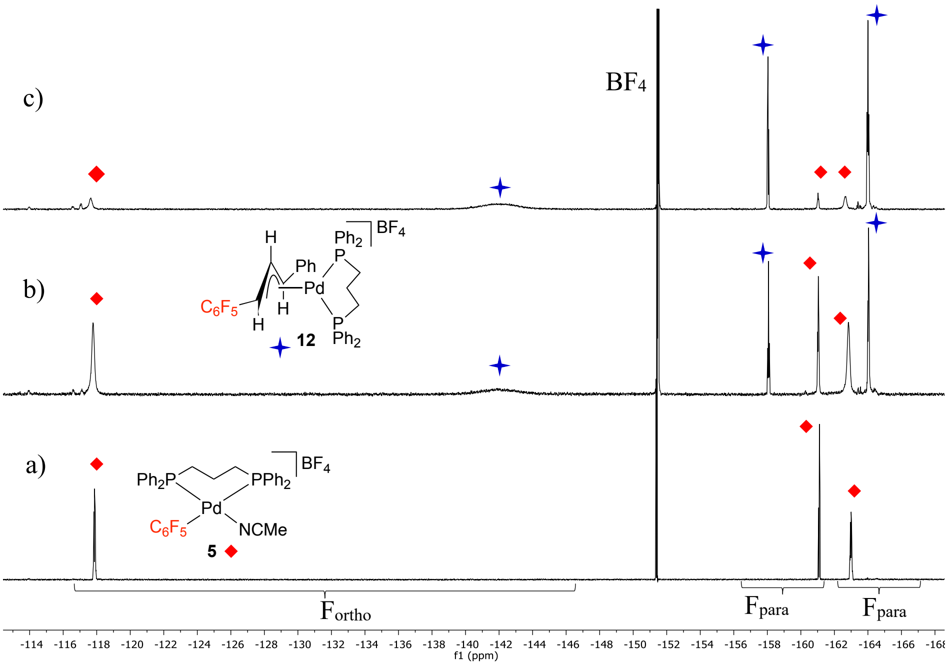

Figure S2. ^{19}F NMR (470.17 MHz, CH_3CN, $(CD_3)_2SO$ capillary) of: a) Complex **5**. b) Complex **5** upon addition of diazoalkane $N_2CH–CH=CHPh$ (**8**) (Pd:**8** = 1:1 mol ratio) c) Sample shown in b) upon addition of an additional equimolar amount of **8** (total mol ratio Pd:**8** = 1:2). (see text, Scheme 3b).

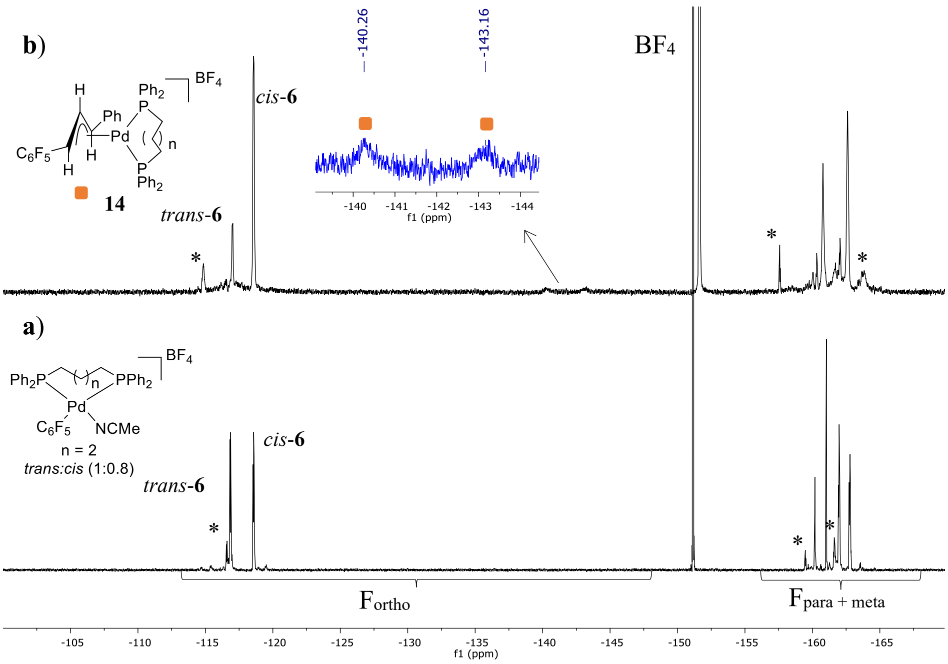

Figure S3. ^{19}F NMR (470.17 MHz, CH_3CN, $(CD_3)_2SO$ capillary) of: a) complex **6**; b) the reaction of complex **6** upon addition of diazoalkane $N_2CH–CH=CHPh$ (**8**) (see text, Scheme 3c). * Signals corresponding to unidentified "[Pd-C_6F_5]" species. Only F_{ortho} signlas are labeled for simplicity.

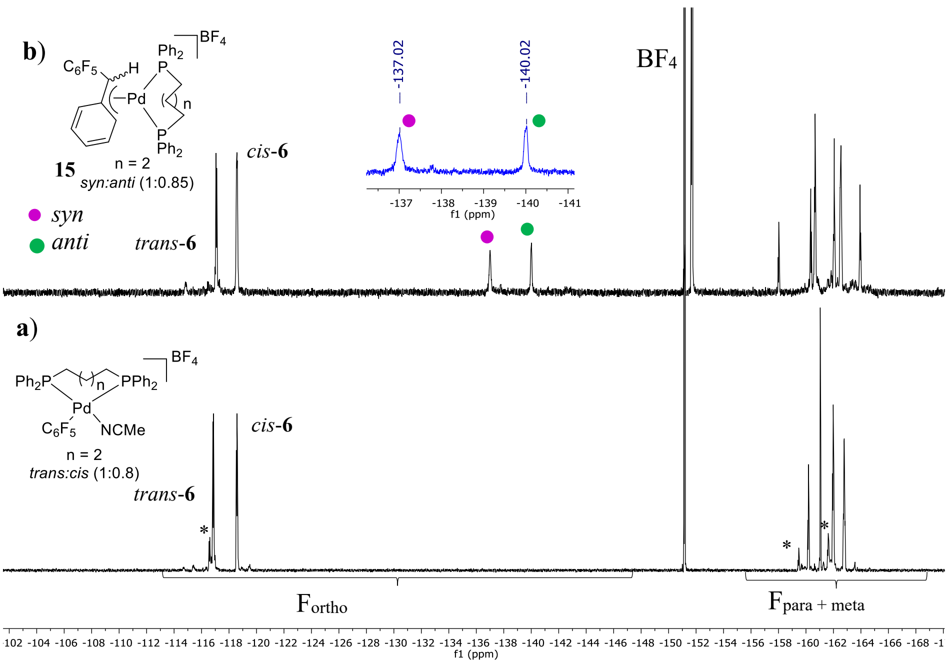

Figure S4. ^{19}F NMR (470.17 MHz, CH_3CN, $(CD_3)_2SO$ capillary) of: a) complex **6**; b) the reaction of complex **6** upon addition of diazoalkane N_2CHPh (**9**) (see text, Scheme 3c). * Signals corresponding to unidentified "[Pd-C_6F_5]" species. Only F_{ortho} signlas are labeled for simplicity.

2. Data for X-Ray molecular structure determinations

Crystals suitable for X-ray analyses were obtained by a) slow diffusion of *n*-hexane layered onto a solution of the complex **12** in $CHCl_3$ at -28 ˚C; b) slow evaporation of the complex **2** in $CHCl_3$. In each case, the crystal was attached to the tip of a glass fiber and transferred to an Agilent Supernova diffractometer with an Atlas CCD area detector. Data collection was performed with Mo Kα radiation (0.71073 Å) at 298 K. Data integration and empirical absorption correction was carried out using the CrysAlisPro program package.[1] The structures were solved by direct methods and refined by full-matrix least squares against F^2 with SHELX,[2] in OLEX2.[3] The non-hydrogen atoms were refined anisotropically and hydrogen atoms were constrained to ideal geometries and refined with fixed isotropic displacement parameters. Refinement of both structures with the restrictions mentioned above proceeded smoothly to give the residuals shown in Table S1.

Two independent molecules were found in the asymmetric unit for **2**. The Olex2 solvent mask was applied because of poorly defined solvent molecules. A solvent mask was calculated and 428 electrons were found in a volume of 1452 cubic angstroms in one void per unit cell. The solvent of crystallization was chloroform and this is consistent with the presence of roughly 2 $CHCl_3$ per formula unit.

Complex **12** presents a positional disorder of the -C_6F_5 and -Ph groups in the allyl-fragment. The structure was refined splitting the positions for each atom with different occupancies. As a consequence, the C-C distances in both aromatic rings and the C-F bond lengths of the -C_6F_5 group are inconsistent with the typical distances of these groups. This positional disorder does not affect the distances and angles of the allylic atoms coordinated to palladium.

The crystal structures have been deposited in the CCDC database: CCDC-2390743 (complex **2**); CCDC-2390734 (complex **12**).

Table S1. Crystal data and structure refinement parameters for complexes **2** and **12**.

Compound number	**2**	**12**
Empirical formula	$C_{67}H_{53}Br_2Cl_3F_{10}P_4Pd_2$	$C_{45}H_{37}BCl_9F_9P_2Pd$
Formula weight	1650.94	1246.94
Temperature/K	298	298
Crystal system	monoclinic	monoclinic
Space group	$P2_1/n$	$P2_1/n$
a/Å	15.0113(2)	13.6190(6)
b/Å	13.1748(3)	14.8420(4)
c/Å	38.0157(8)	26.4643(10)

α/°	90	90
β/°	91.428(2)	96.984(4)
γ/°	90	90
Volume/$Å^3$	7516.1(3)	5309.6(3)
Z	4	4
ρ_{calc}g/cm^3	1.459	1.560
μ/mm^{-1}	1.794	0.926
F(000)	3272.0	2488.0
Crystal size/mm^3	0.626 × 0.479 × 0.32	0.661 × 0.19 × 0.135
Radiation	Mo Kα (λ = 0.71073)	Mo Kα (λ = 0.71073)
2Θ range for data collection/°	6.546 to 59.426	6.826 to 59.332
Index ranges	-20 ≤ h ≤ 15, -17 ≤ k ≤ 14, -52 ≤ l ≤ 52	-12 ≤ h ≤ 18, -20 ≤ k ≤ 19, -36 ≤ l ≤ 36
Reflections collected	41516	29444
Independent reflections	17849 [R_{int} = 0.0274, R_{sigma} = 0.0452]	12962 [R_{int} = 0.0338, R_{sigma} = 0.0564]
Data/restraints/parameters	17849/0/793	12962/0/625
Goodness-of-fit on F^2	1.015	1.064
Final R indexes [I>=2σ (I)]	R_1 = 0.0433, wR_2 = 0.0861	R_1 = 0.0840, wR_2 = 0.2388
Final R indexes [all data]	R_1 = 0.0671, wR_2 = 0.0954	R_1 = 0.1404, wR_2 = 0.2870
Largest diff. peak/hole / e $Å^{-3}$	0.74/-0.95	1.02/-0.74

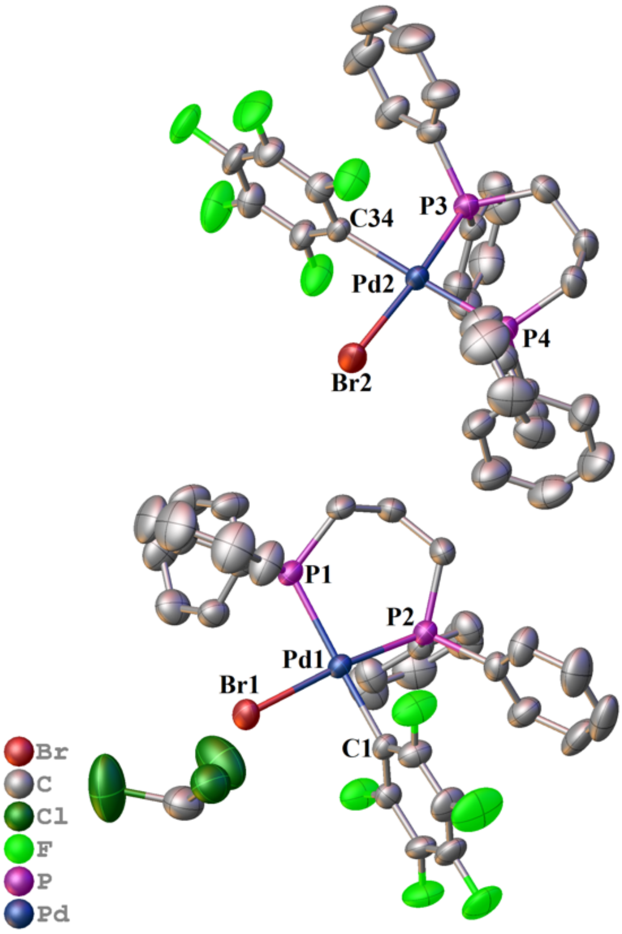

Figure S5. X-ray molecular structure of **2** (ORTEP 40% probability ellipsoids). Hydrogen atoms are omitted for clarity. The two independent molecules are shown.

Table S2. Selected bond lengths (Å) and angles (º) for complex **2** (numbering scheme in Figure S5).

Pd(1)-Br(1)	2.4756(4)	Pd(2)-Br(2)	2.4752(4)
Pd(1)-P(1)	2.3286(9)	Pd(2)-P(3)	2.2500(8)
Pd(1)-P(2)	2.2483(8)	Pd(2)-P(4)	2.3293(9)
Pd(1)-C(1)	2.062(3)	Pd(2)-C(34)	2.056(3)
P(2)-Pd(1)-P(1)	93.44(3)	P(3)-Pd(2)-P(4)	94.23(3)
C(1)-Pd(1)-Br(1)	88.28(8)	C(34)-Pd(2)-Br(2)	87.36(8)

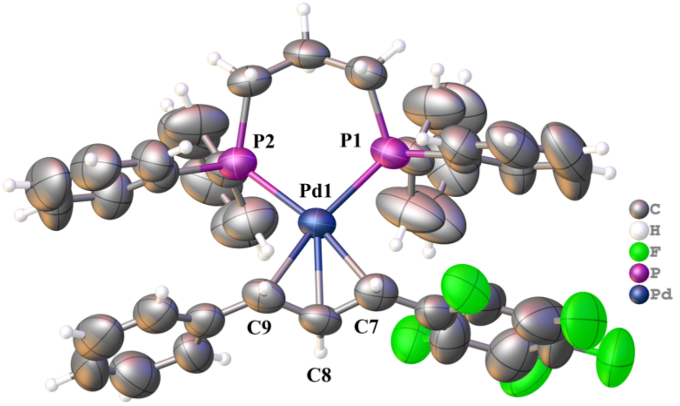

Figure S6. X-ray molecular structure of **12** (ORTEP 40% probability ellipsoids). Solvent molecules ($CHCl_3$) and the BF_4^- anion are omitted for clarity.

Table S3. Selected bond lengths (Å) and angles (º) for complex **12** (numbering scheme in Figure S6).

Pd(1)-P(2)	2.3035(17)	P(2)-Pd(1)-P(1)	93.10(6)
Pd(1)-P(1)	2.3057(16)	C(7)-Pd(1)-C(9)	66.9(3)
Pd(1)-C(9)	2.229(6)	C(7)-C(8)-C(9)	122.2(7)
Pd(1)-C(8)	2.196(6)		
Pd(1)-C(7)	2.226(7)		
C(9)-C(8)	1.397(10)		
C(8)-C(7)	1.406(10)		

3. Selected spectra

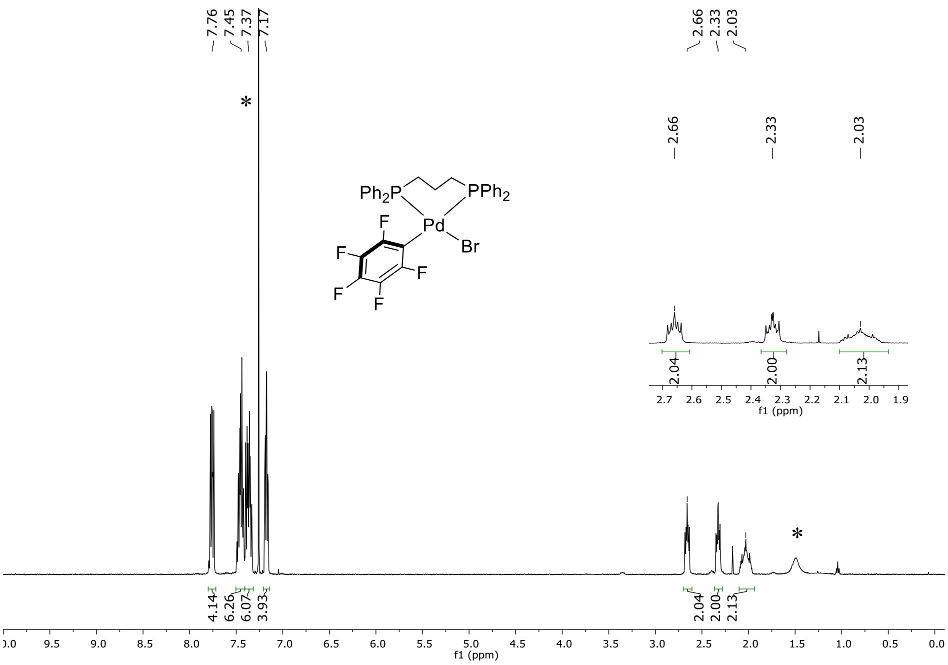

Figure S7. ^{1}H NMR (499.73 MHz, $CDCl_3$) of **[PdBr(C_6F_5)(dppp)]** (**2**) at 298 K. * Signals corresponding to the solvent (chloroform and water).

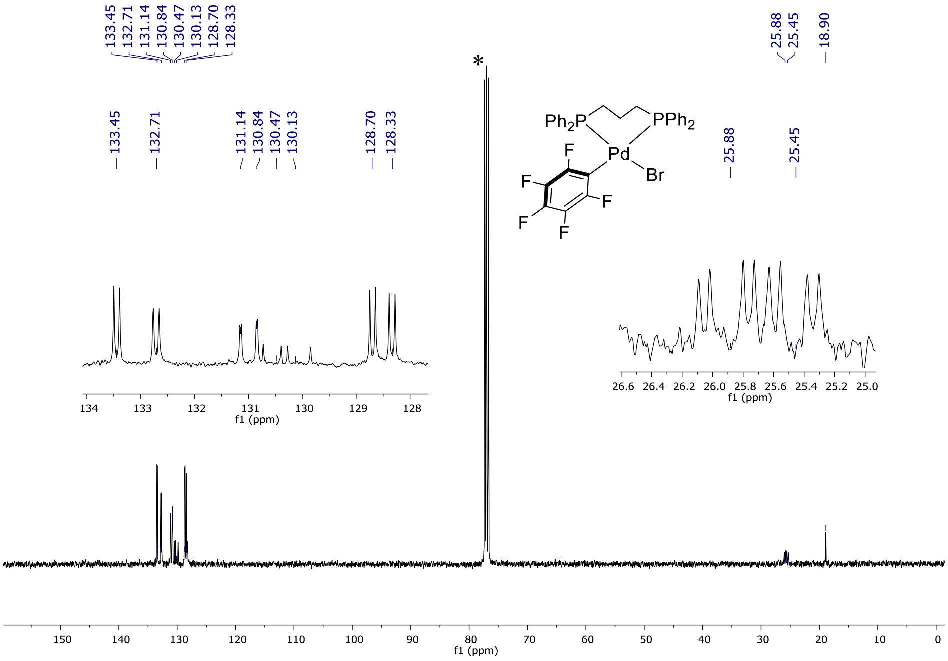

Figure S8. $^{13}C\{^{1}H\}$ NMR (125.67 MHz, $CDCl_3$) of **[PdBr(C_6F_5)(dppp)]** (**2**) at 298 K. * Signals corresponding to the solvent (chloroform).

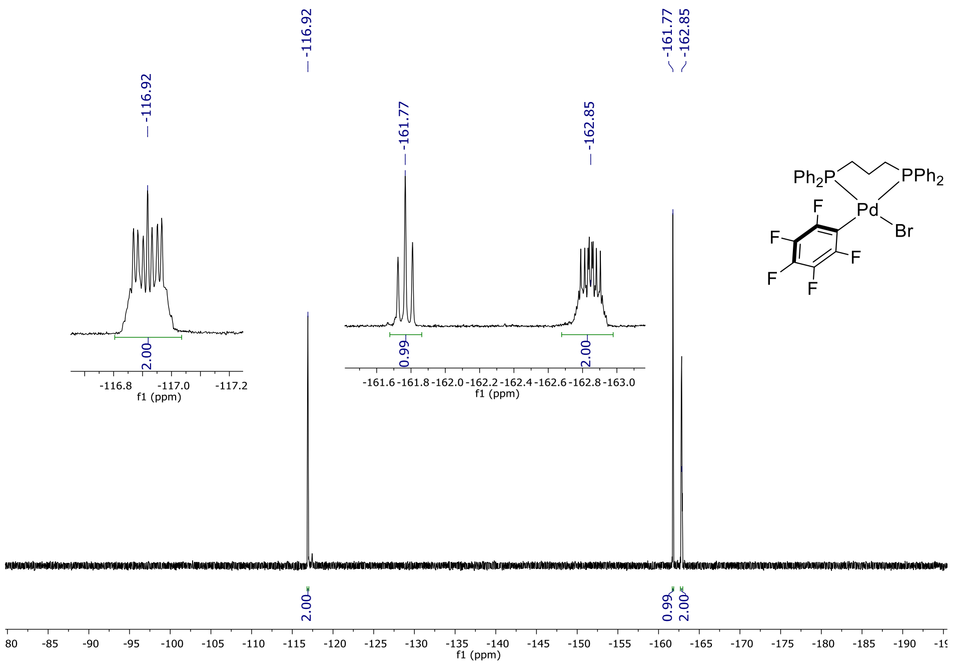

Figure S9. ^{19}F NMR (470.17 MHz, $CDCl_3$) of **[PdBr(C_6F_5)(dppp)]** (**2**) at 298 K.

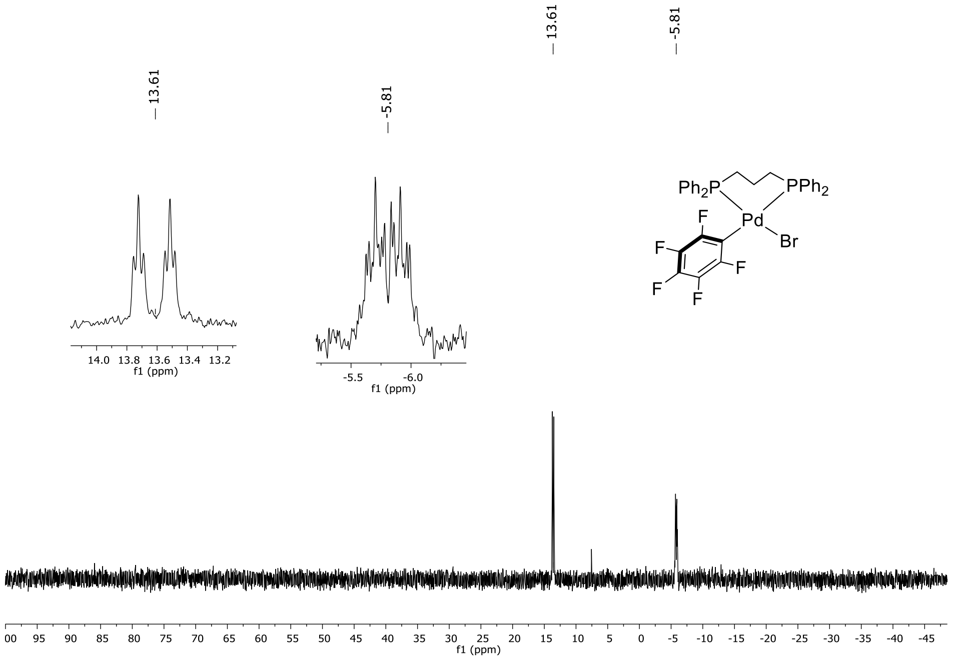

Figure S10. ^{31}P{^{1}H} NMR (202.31 MHz, $CDCl_3$) of **[PdBr(C_6F_5)(dppp)]** (**2**) at 298 K.

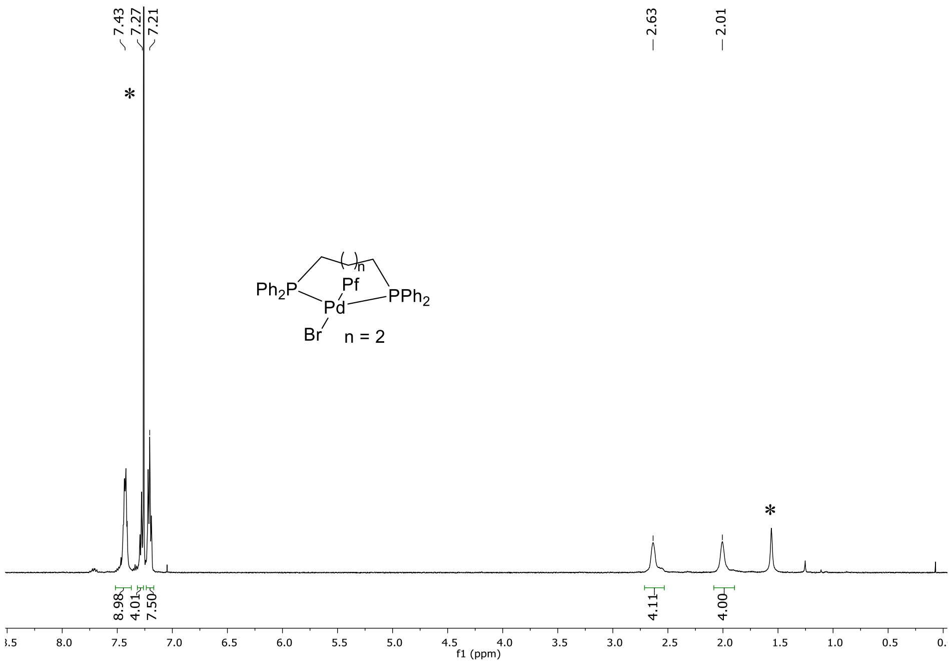

Figure S11 ^{1}H NMR (499.72 MHz, $CDCl_3$) of *trans*-**[PdBr(C_6F_5)(dppb)]** (**3**) at 298 K. * Signals corresponding to the solvent (chloroform and water).

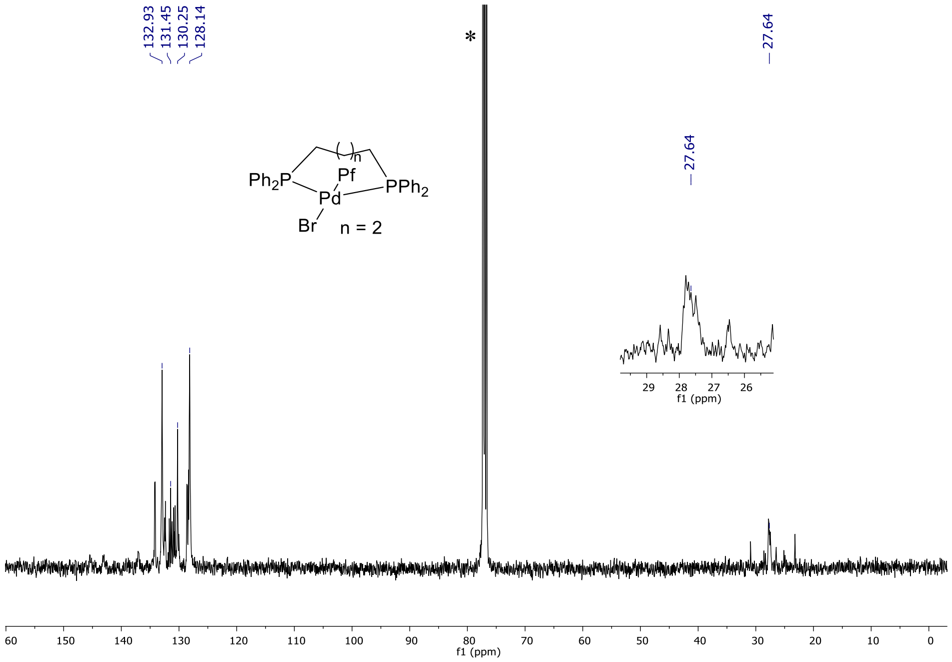

Figure S12. $^{13}C\{^{1}H\}$ NMR (125.67 MHz, $CDCl_3$) of *trans*-**[PdBr(C_6F_5)(dppb)]** (**3**) at 298 K. * Signals corresponding to the solvent (chloroform).

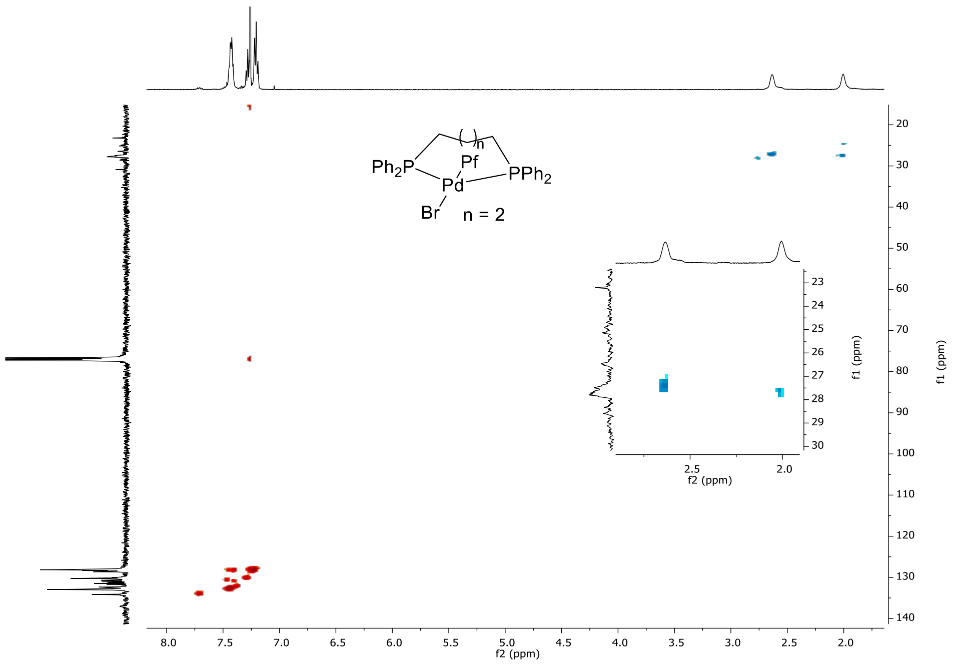

Figure S13. ^{1}H-^{13}C gHSQCAD NMR of *trans*-**[PdBr(C_6F_5)(dppb)]** (**3**) at 298 K.

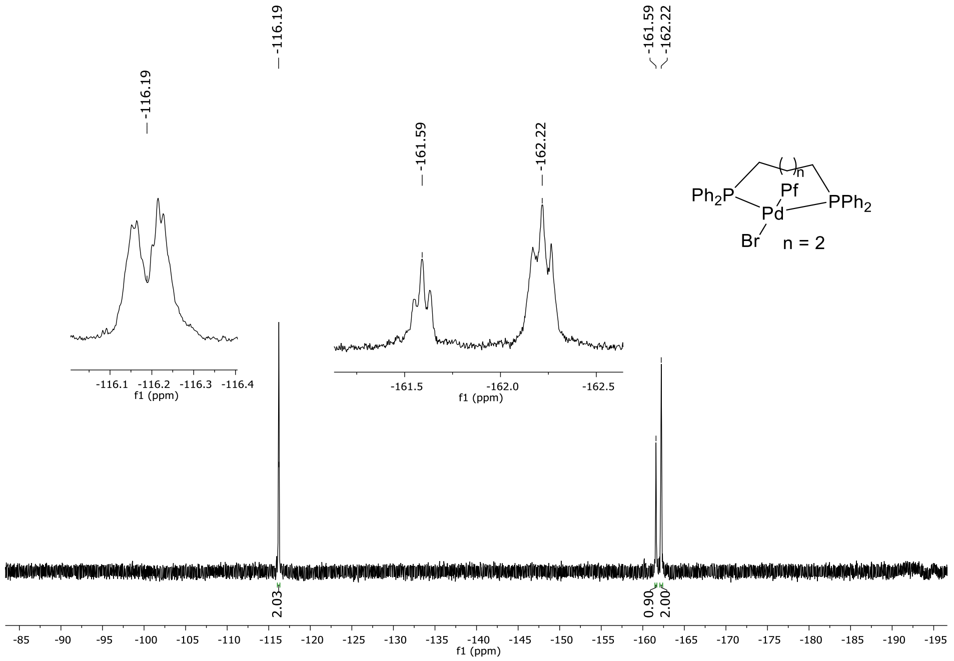

Figure S14. ^{19}F NMR (470.17 MHz, $CDCl_3$) of *trans*-**[PdBr(C_6F_5)(dppb)]** (**3**) at 298 K.

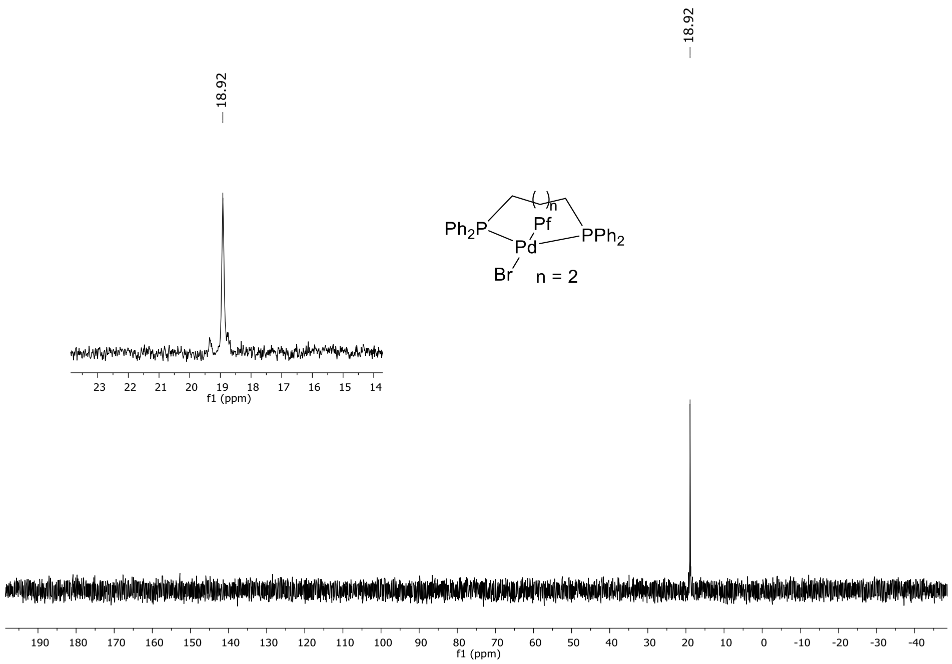

Figure S15. ${}^{31}P\{{}^{1}H\}$ NMR (202.31 MHz, $CDCl_3$) of *trans*-**[PdBr(C_6F_5)(dppb)]** (**3**) at 298 K.

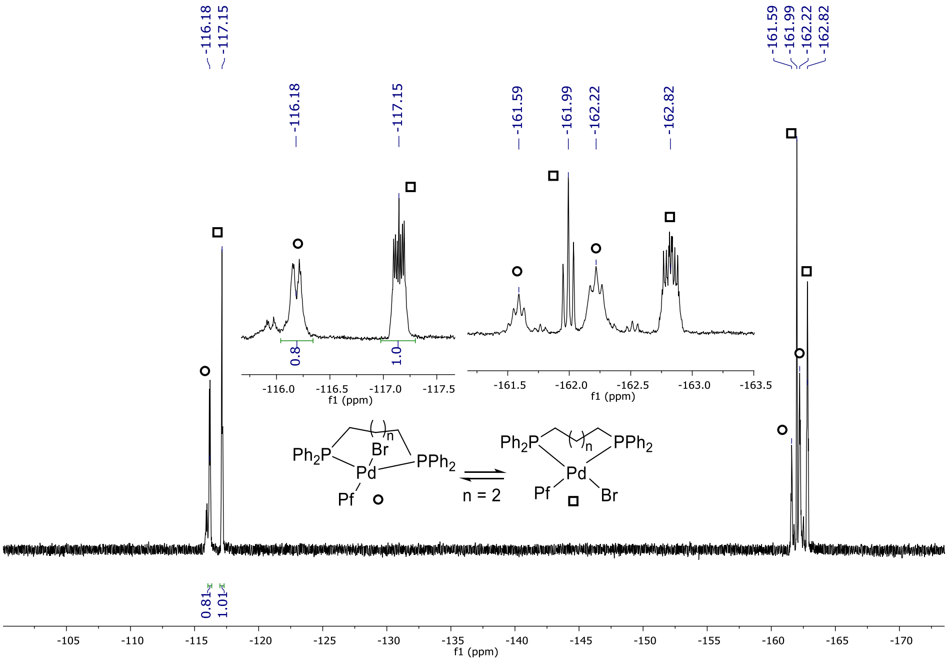

Figure S16. ${}^{19}F$ NMR (470.17 MHz, $CDCl_3$) of a mixture *cis*:*trans* (1:0.8) of **[PdBr(C_6F_5)(dppb)]** (**3**) after 48 h at 298 K.

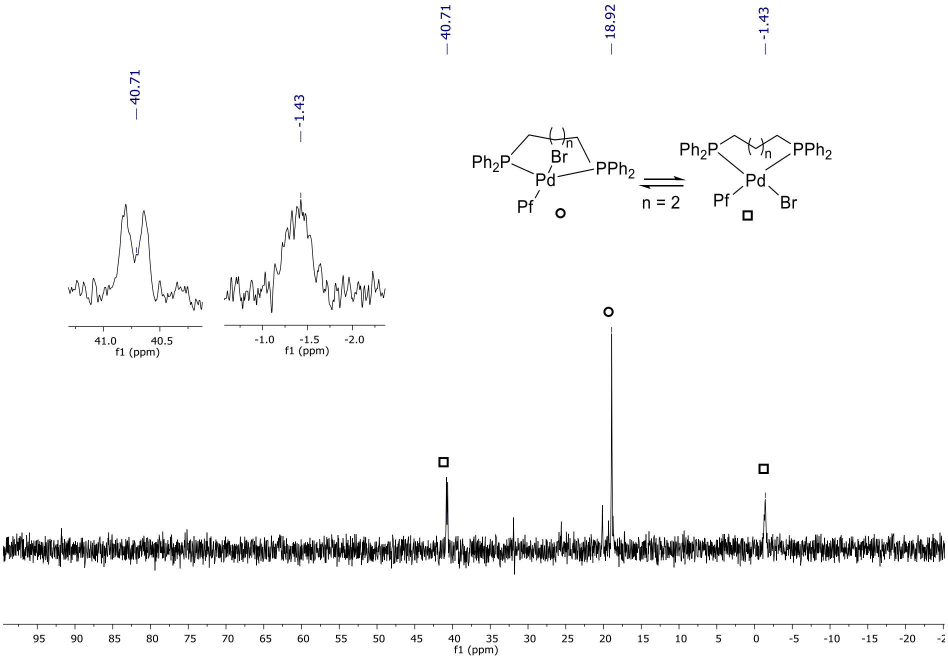

Figure S17. $^{31}P\{^{1}H\}$ NMR (202.31, MHz, $CDCl_3$) of a mixture *cis*:*trans* (1:0.8) of **[PdBr(C_6F_5)(dppb)]** (**3**) after 48 h at 298 K.

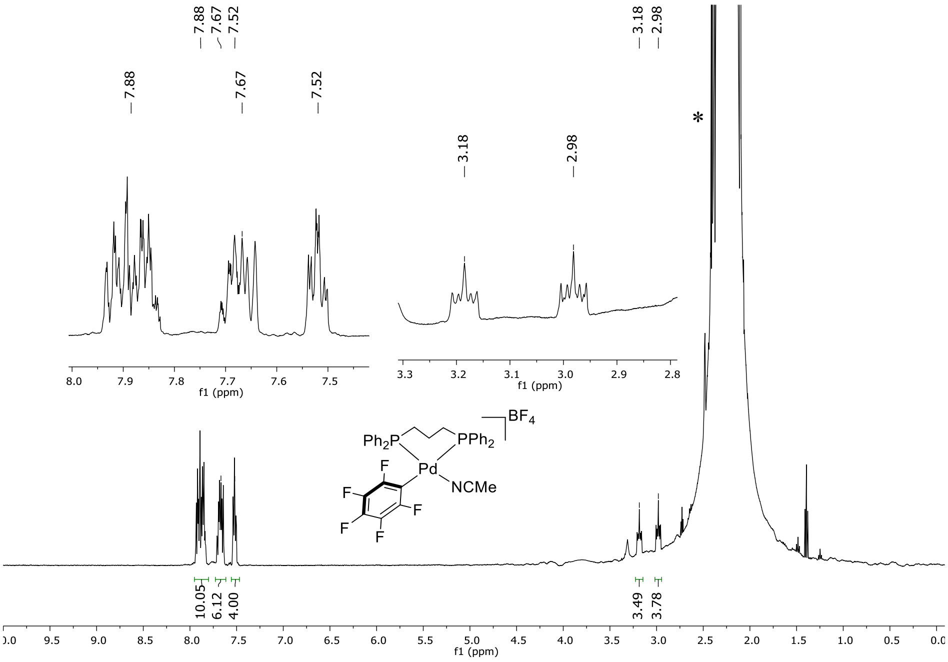

Figure S18. ^{1}H NMR (499.73 MHz, CH_3CN, $(CD_3)_2SO$ capillary) of **[Pd(C_6F_5)(dppp)(NCMe)](BF_4)** (**5**) at 298 K. * Signals corresponding to the solvent (acetonitrile).

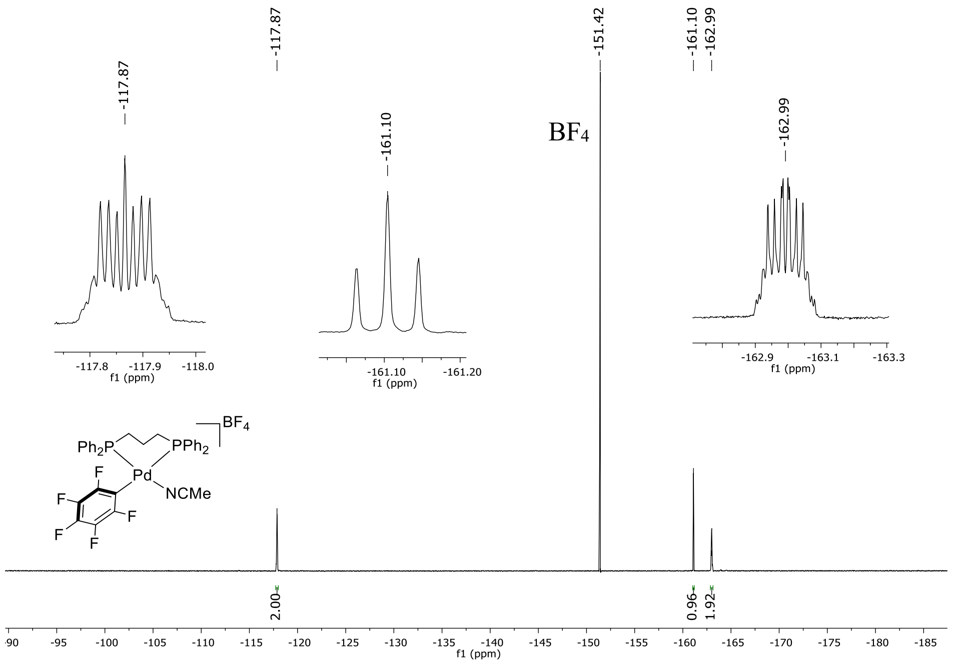

Figure S19. ^{19}F NMR (470.17 MHz, CH_3CN, $(CD_3)_2SO$ capillary) of **[Pd(C$_6$F$_5$)(dppp)(NCMe)](BF$_4$)** (**5**) at 298 K.

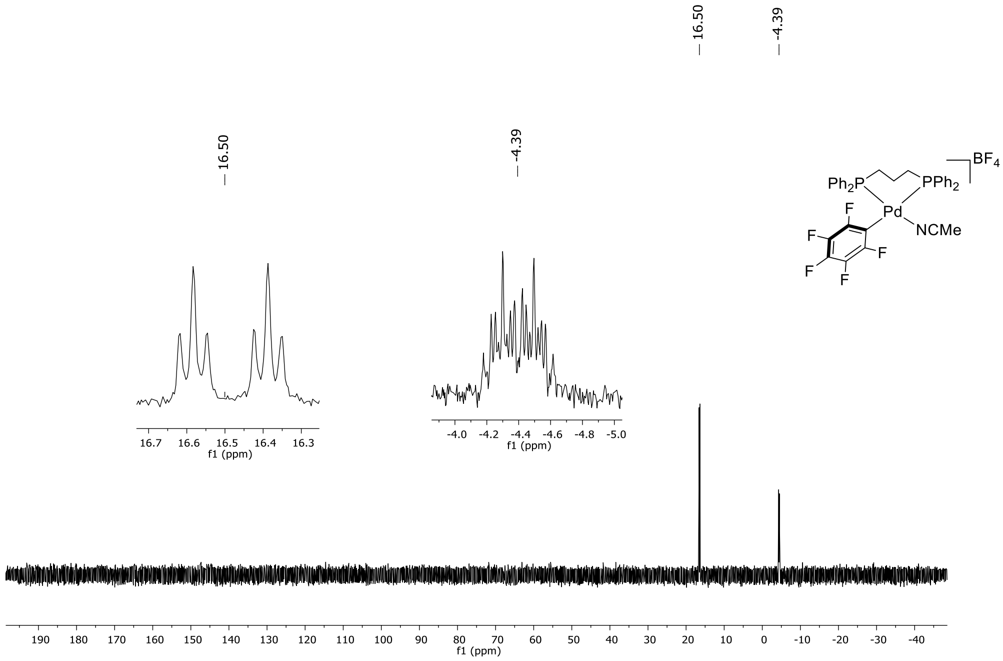

Figure S20. $^{31}P\{^1H\}$ NMR (202.31, MHz, CH_3CN, $(CD_3)_2SO$ capillary) of **[Pd(C$_6$F$_5$)(dppp)(NCMe)](BF$_4$)** (**5**) at 298 K.

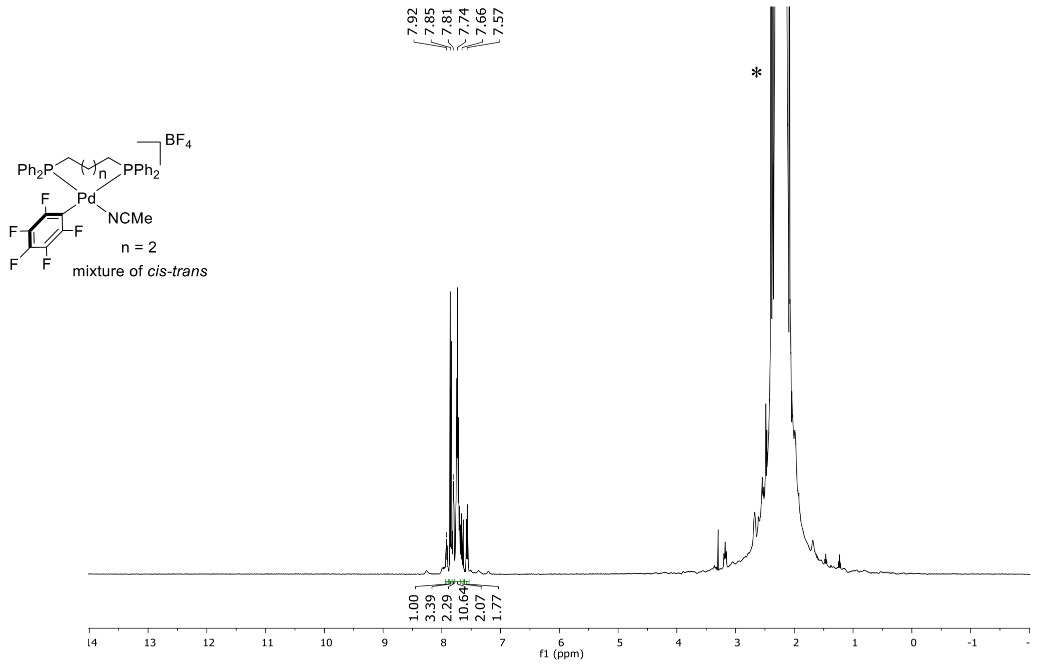

Figure S21. ^{1}H NMR (499.73 MHz, CH_3CN, $(CD_3)_2SO$ capillary) of a mixture of *cis*:*trans* (0.8:1) **[Pd(C_6F_5)(dppb)(NCMe)](BF_4)** (**6**) at 298 K. * Signals corresponding to the solvent (acetonitrile).

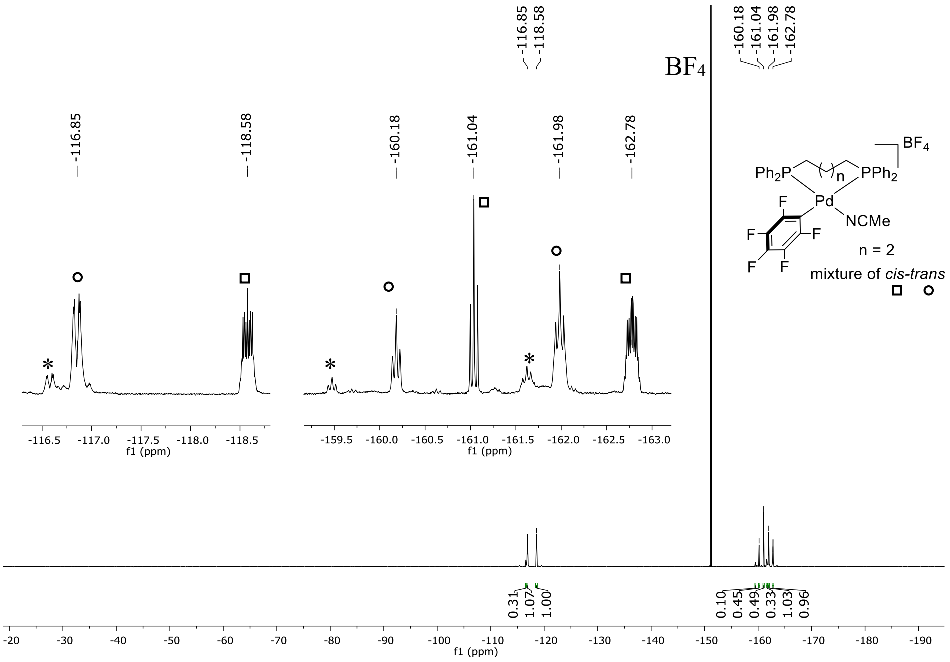

Figure S22. ^{19}F NMR (470.17 MHz, CH_3CN,$(CD_3)_2SO$ capillary) of a mixture *cis*:*trans* (0.8:1) of **[Pd(C_6F_5)(dppb)(NCMe)](BF_4)** (**6**) at 298 K. * Signals corresponding to Pd-complex reorganization.

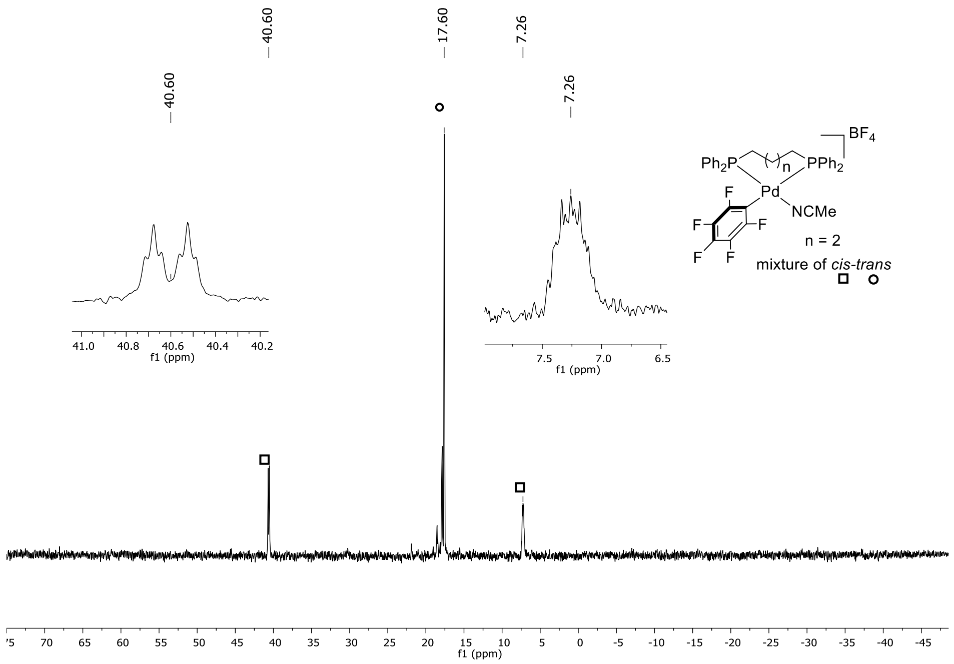

Figure S23. $^{31}P\{^{1}H\}$ NMR (202.31, MHz, CH_3CN,$(CD_3)_2SO$ capillary) of a mixture of **[Pd(C_6F_5)(dppb)(NCMe)](BF_4) (6)** *cis*:*trans* (0.8:1) at 298 K. * Signals corresponding to the starting Pd-complex (*cis*-*trans* mixture).

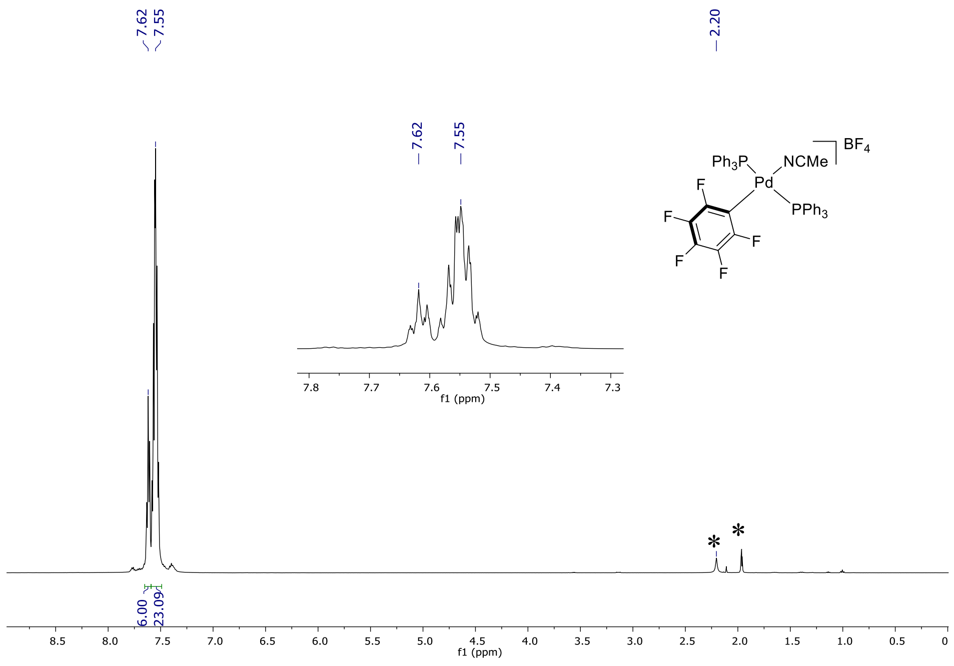

Figure S24. ^{1}H NMR (499.73 MHz, CD_3CN) of **[Pd(C_6F_5)($NCCH_3$)(PPh_3)$_2$](BF_4)** (**7**) at 298 K. * Signals corresponding to the solvent (acetonitrile and water).

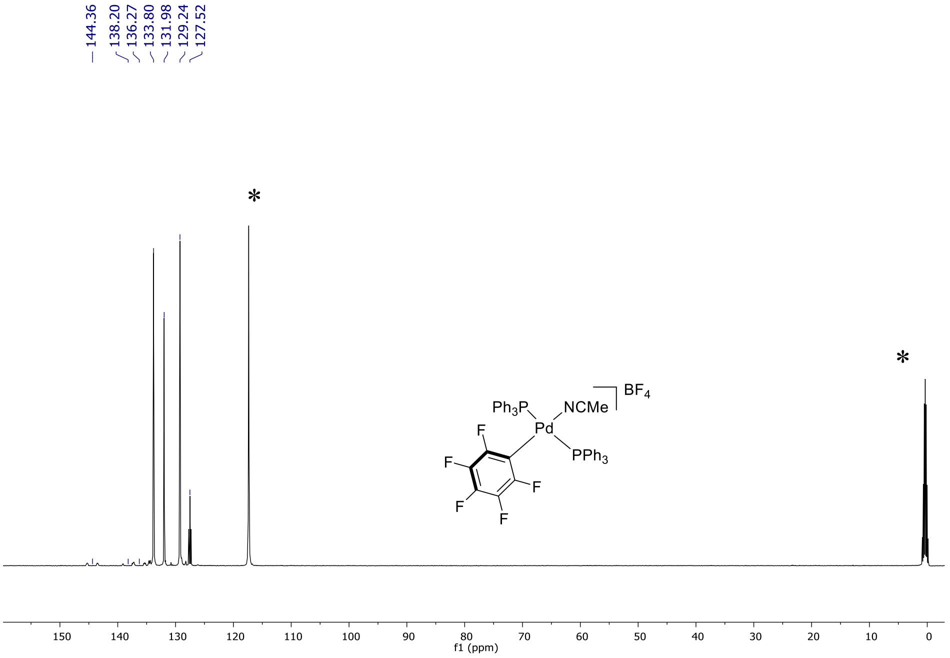

Figure S25. $^{13}C\{^1H\}$ NMR (125.67 MHz, CD_3CN) of **$[Pd(C_6F_5)(NCCH_3)(PPh_3)_2](BF_4)$** (**7**) at 298 K. * Signals corresponding to the solvent (acetonitrile).

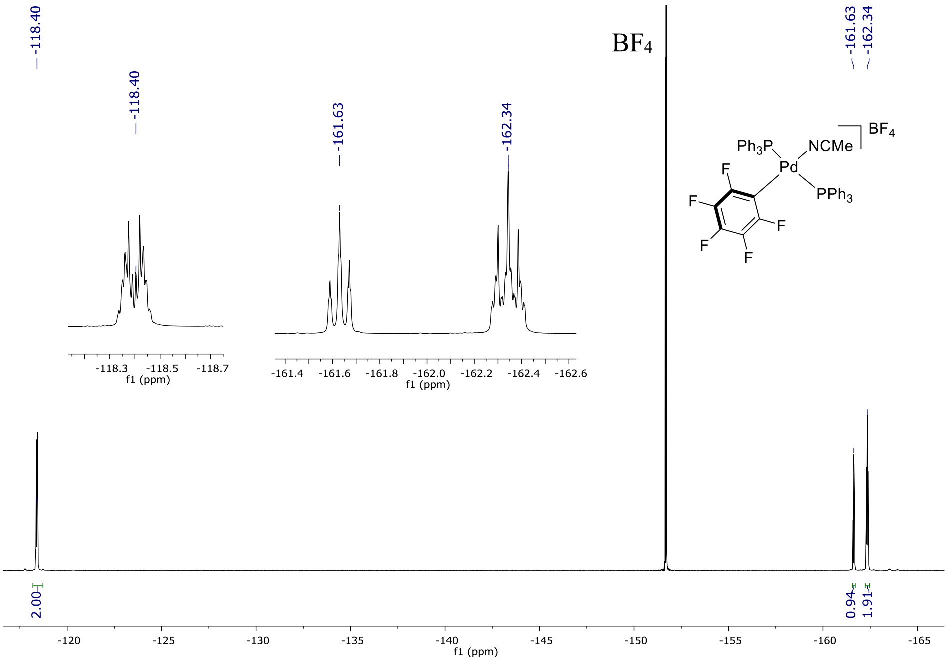

Figure S26. ^{19}F NMR (470.17 MHz, CD_3CN) of **$[Pd(C_6F_5)(NCCH_3)(PPh_3)_2](BF_4)$** (**7**) at 298 K.

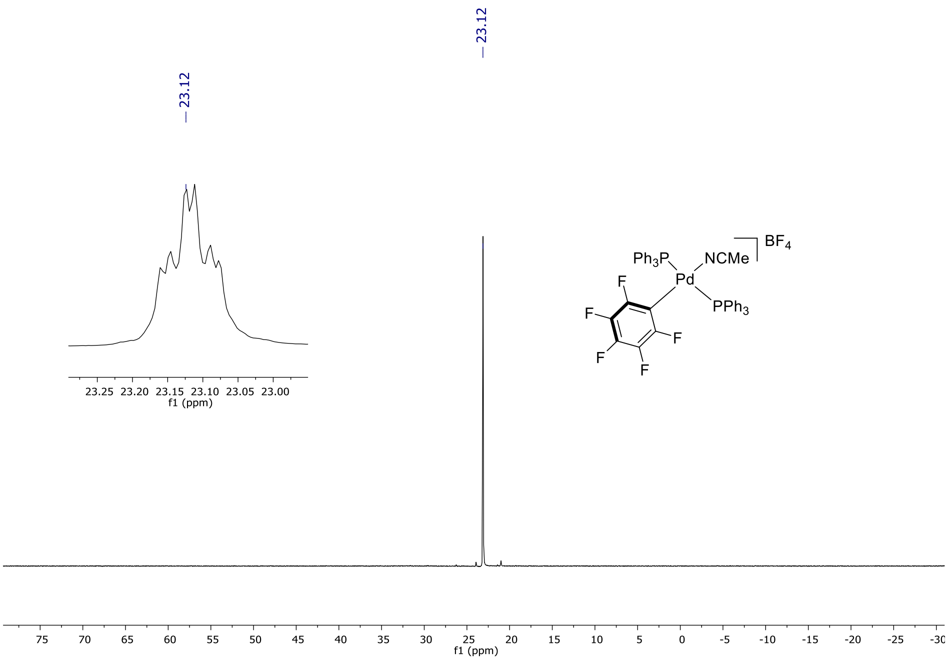

Figure S27. ^{31}P NMR (202.29 MHz, CD_3CN) of **[Pd(C_6F_5)($NCCH_3$)(PPh_3)$_2$](BF_4)** (**7**) at 298 K.

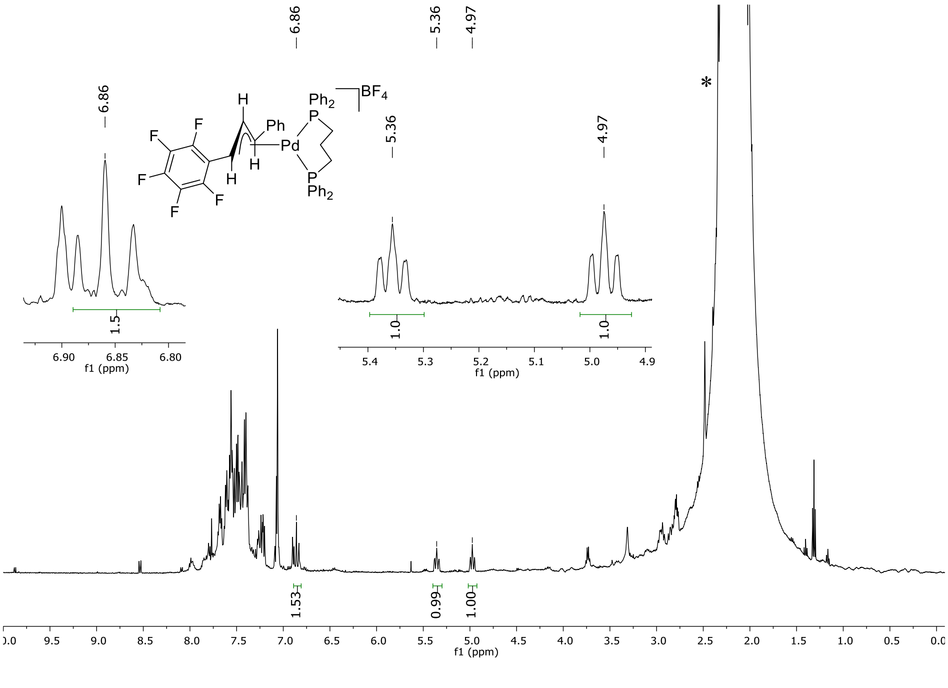

Figure S28. ^{1}H NMR (499.73 MHz, CH_3CN, $(CD_3)_2SO$ capillary) of **[Pd(dppp)(η^3-Ph-CH-CH-CH-C_6F_5)](BF_4)** (**12**) at 298 K. * Signals corresponding to the solvent (acetonitrile).

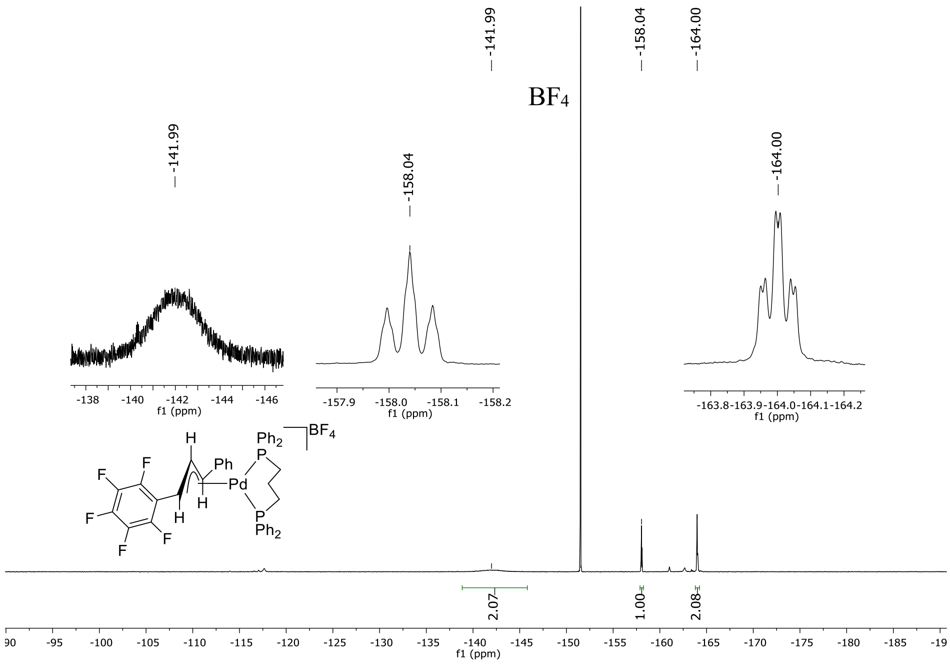

Figure S29. ^{19}F NMR (470.17 MHz, $CH_3CN,(CD_3)_2SO$ capillary) of **[Pd(dppp)(η^3-Ph-CH-CH-CH-C_6F_5)](BF_4)** (**12**) at 298 K.

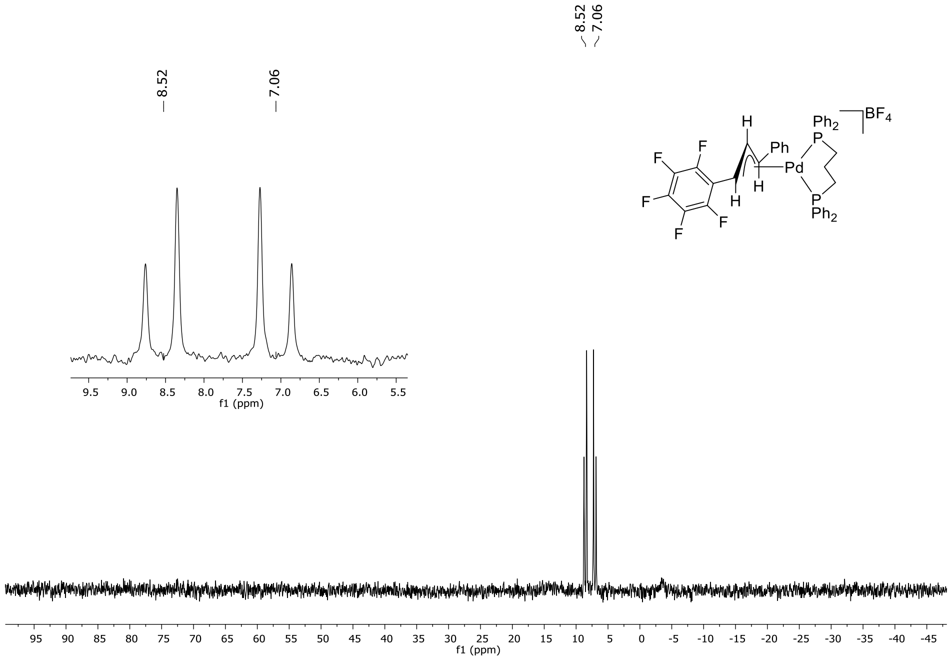

Figure S30. $^{31}P\{^1H\}$ NMR (202.31, MHz, $CH_3CN,(CD_3)_2SO$ capillary) of **[Pd(dppp)(η^3-Ph-CH-CH-CH-C_6F_5)](BF_4)** (**12**) at 298 K.

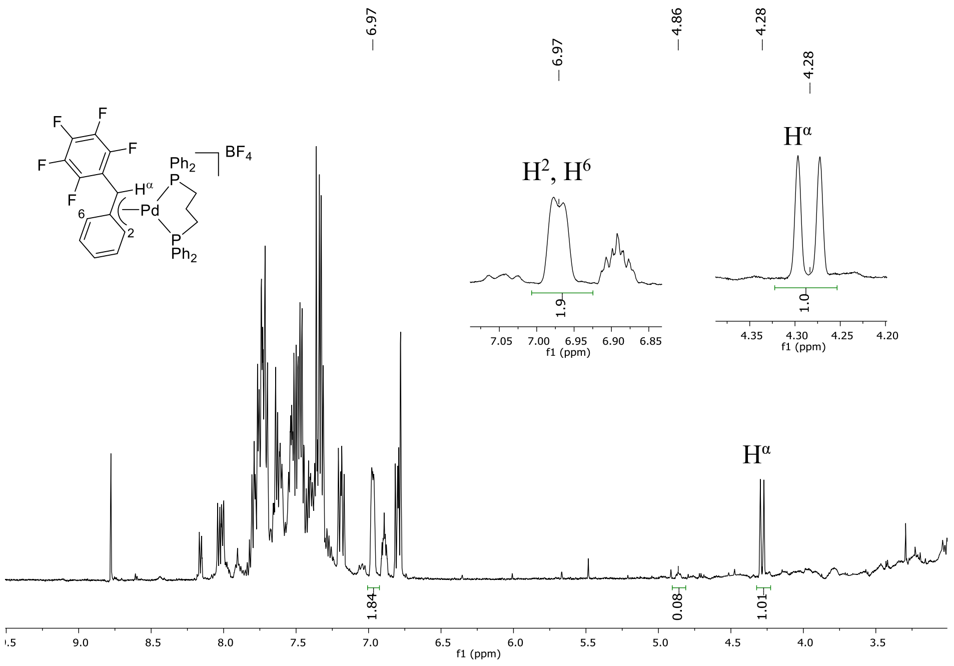

Figure S31. ^{1}H NMR (499.73 MHz, CH_3CN, $(CD_3)_2SO$ capillary) of **[Pd(dppp)(η^3-Ph-CH-C_6F_5)](BF_4) (13)** at 298 K. Signal of acetonitrile has been omitted for clarity.

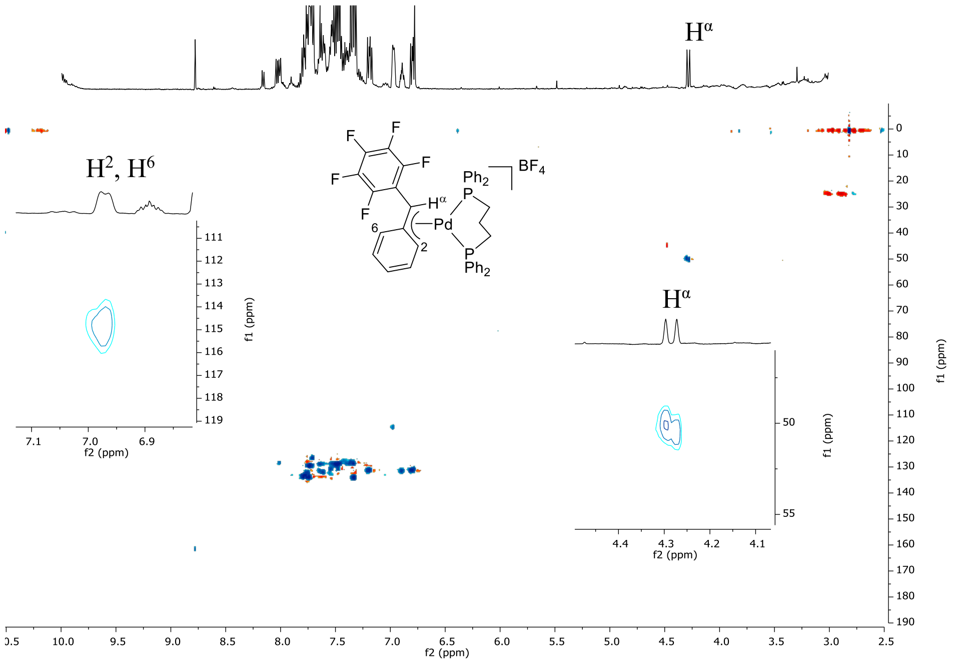

Figure S32. ^{1}H-^{13}C gHSQCAD NMR (499.73 MHz, CH_3CN, $(CD_3)_2SO$ capillary) of **[Pd(dppp)(η^3-Ph-CH-C_6F_5)](BF_4) (13)** at 298 K.

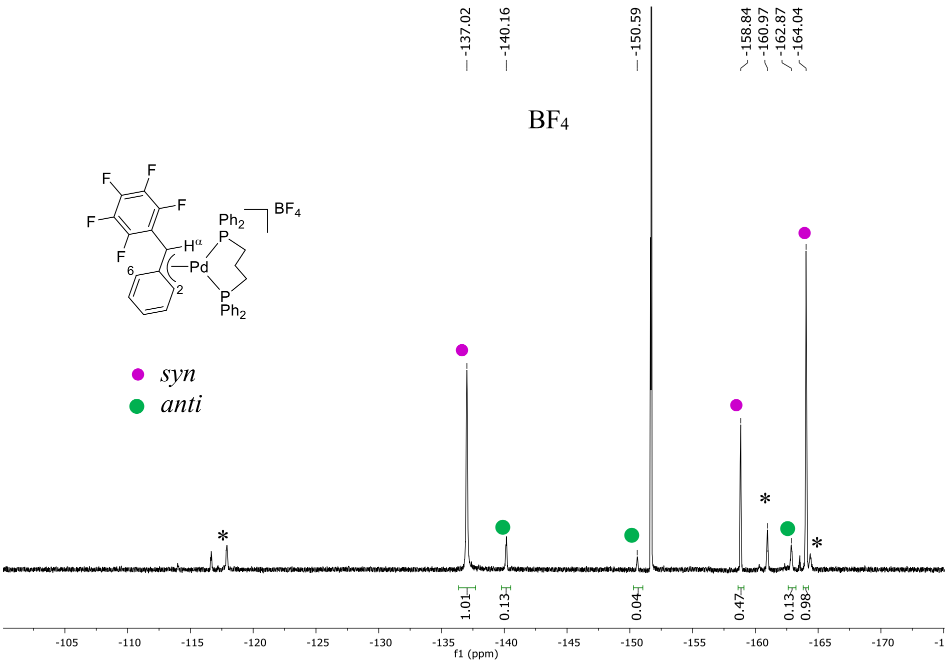

Figure S33. ^{19}F NMR (470.17 MHz, CH_3CN,$(CD_3)_2SO$ capillary) of **[Pd(dppp)(η^3-Ph-CH- C_6F_5)](BF_4)** (**13**) at 298 K. * Signals corresponding to the solvento acetonitrile complex (**5**) and a minor Pd reorganization complex.

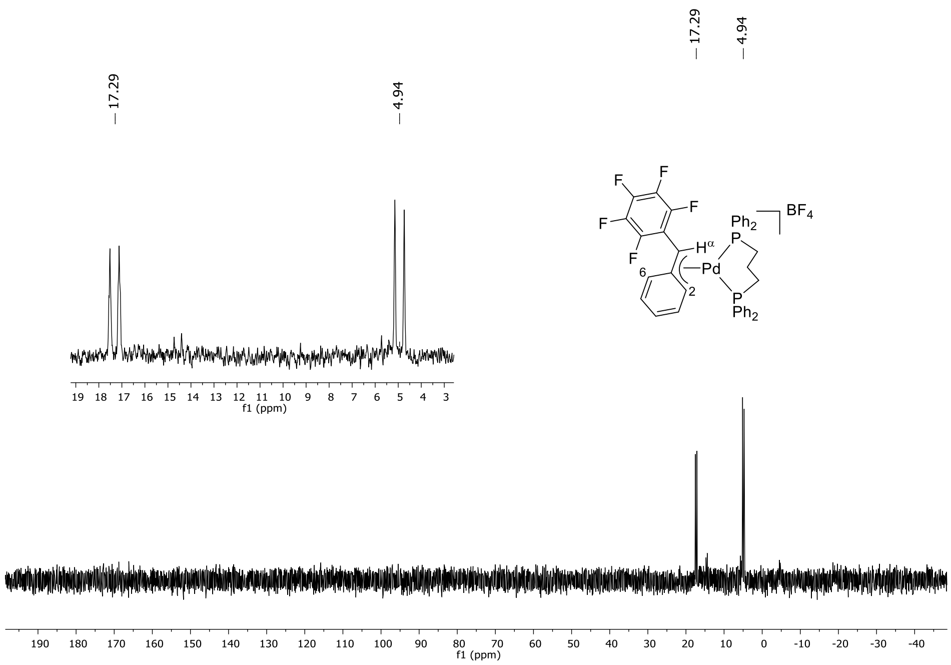

Figure S34. $^{31}P\{^1H\}$ NMR (202.31, MHz, CH_3CN,$(CD_3)_2SO$ capillary) of **[Pd(dppp)(η^3-Ph-CH-C_6F_5)](BF_4)** (**13**) at 298 K.

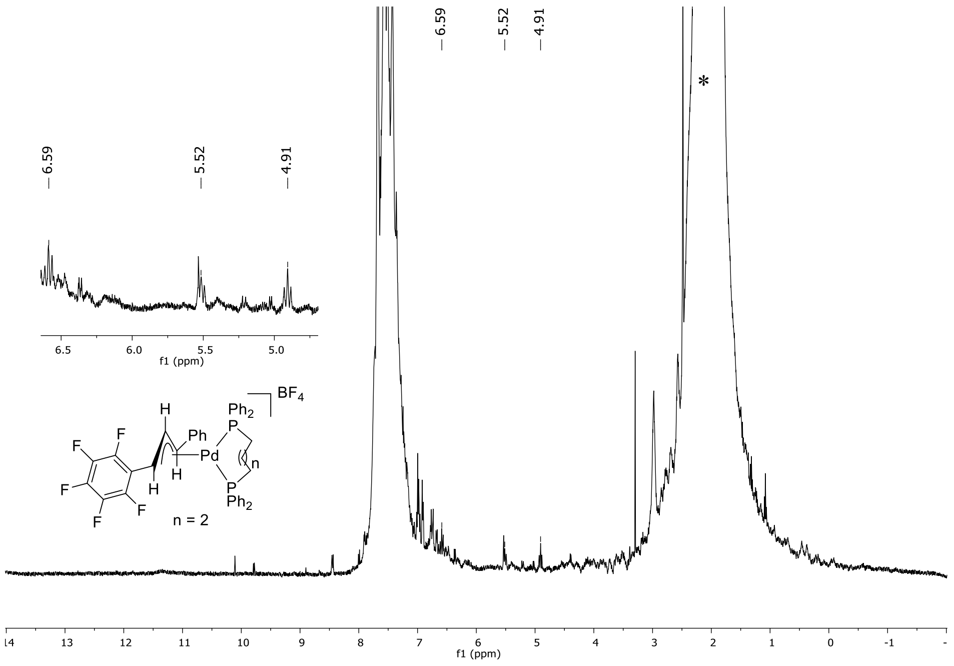

Figure S35. ^{1}H NMR (499.73 MHz, CH_3CN, $(CD_3)_2SO$ capillary) of a solution containing a small amount of **[Pd(dppb)(η^3-Ph-CH-CH-CH-C_6F_5)](BF_4)** (**14**) at 298 K. * Signals corresponding to the solvent (acetonitrile).

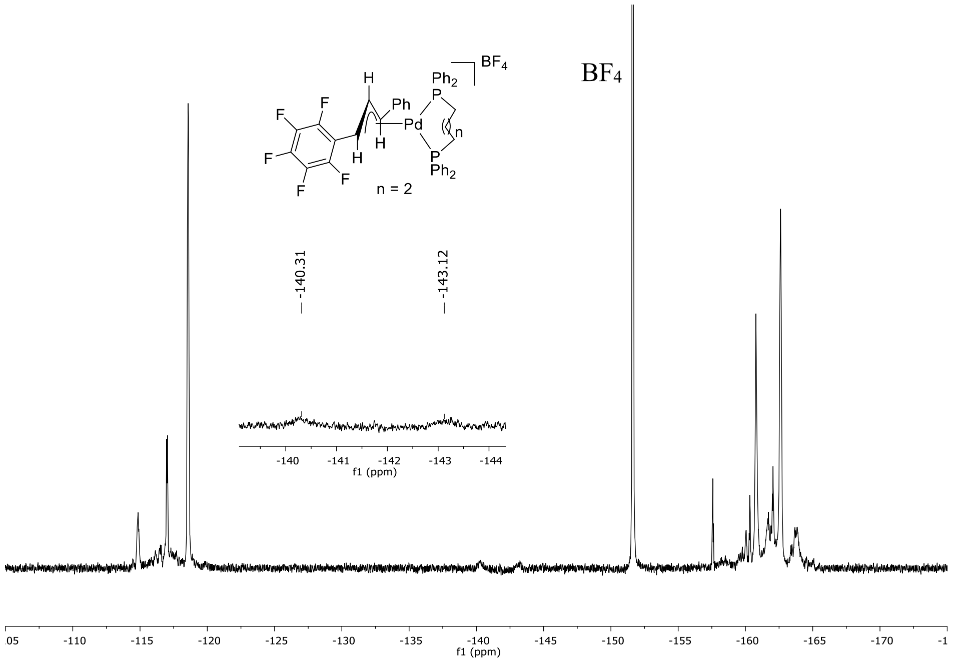

Figure S36. ^{19}F NMR (470.17 MHz, CH_3CN, $(CD_3)_2SO$ capillary) of a solution containing a small amount of **[Pd(dppb)(η^3-Ph-CH-CH-CH-C_6F_5)](BF_4)** (**14**) at 298 K.

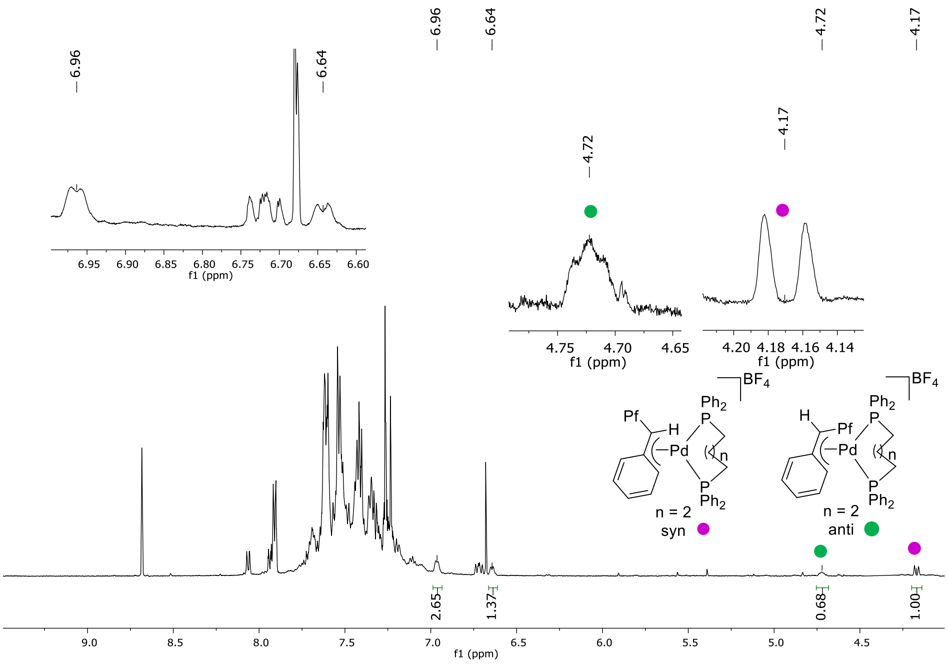

Figure S37. ^{1}H NMR (499.73 MHz, CH_3CN, $(CD_3)_2SO$ capillary) of a solution containing **[Pd(dppb)(η^3-Ph-CH-C_6F_5)](BF_4) (15)** at 298 K. * Signals for acetonitrile has been omitted for clarity.

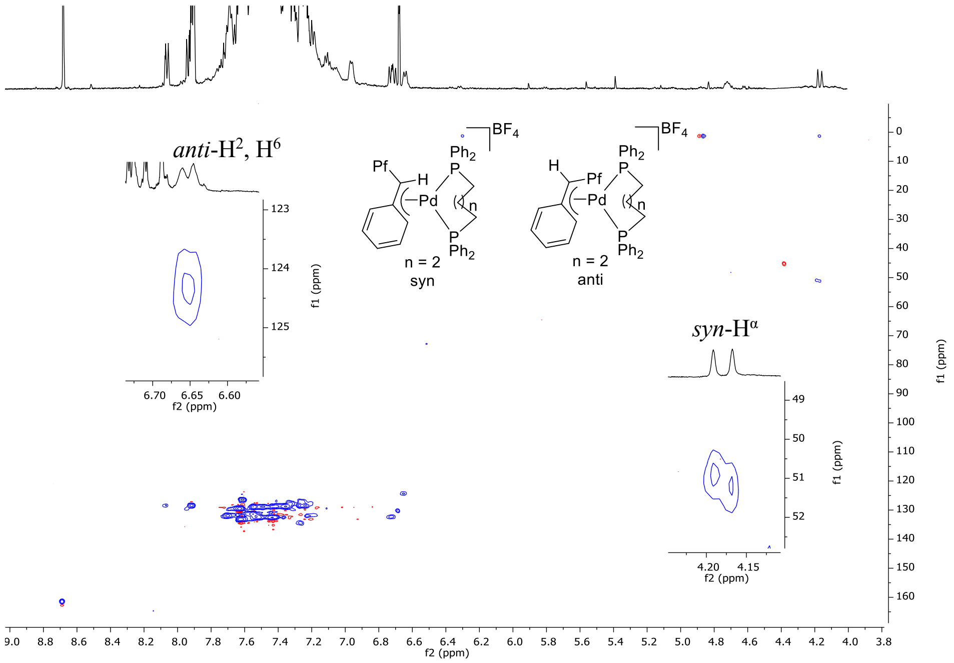

Figure S38.^{1}H-^{13}C gHSQCAD NMR of a solution containing **[Pd(dppb)(η^3-Ph-CH-C_6F_5)](BF_4) (15)** at 298 K.

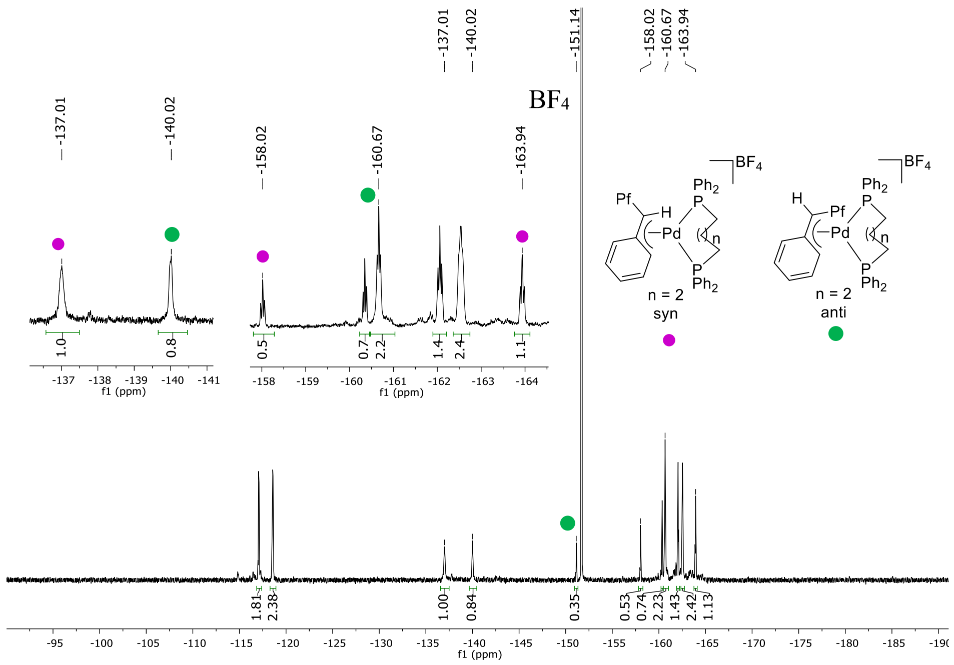

Figure S39. ^{19}F NMR (470.17 MHz, CH_3CN, $(CD_3)_2SO$ capillary) of a solution containing **[Pd(dppb)(η^3-Ph-CH-C_6F_5)](BF_4) (15)** at 298 K.

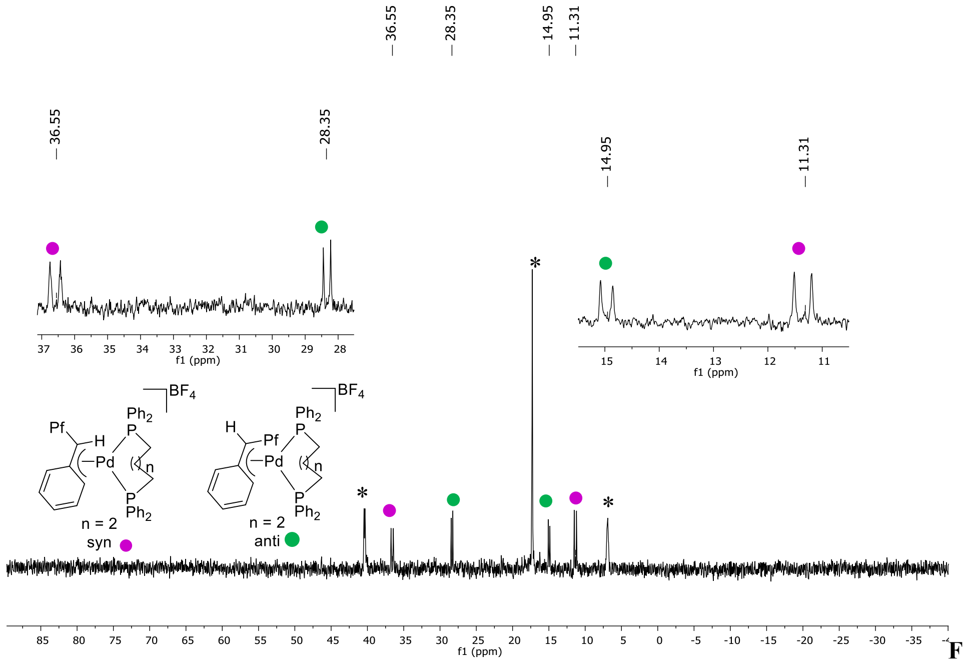

Figure S40. $^{31}P\{^1H\}$ NMR (202.31, MHz, CH_3CN, $(CD_3)_2SO$ capillary) of a solution containing **[Pd(dppb)(η^3-Ph-CH-C_6F_5)](BF_4) (15)** at 298 K. * Signals corresponding to the starting *cis-trans* **6** mixture.

4. Computational Details.

4.1. Selected figures and geometrical parameters.

Relative stabilities of syn and anti isomers in the η^3-benzylic complexes.

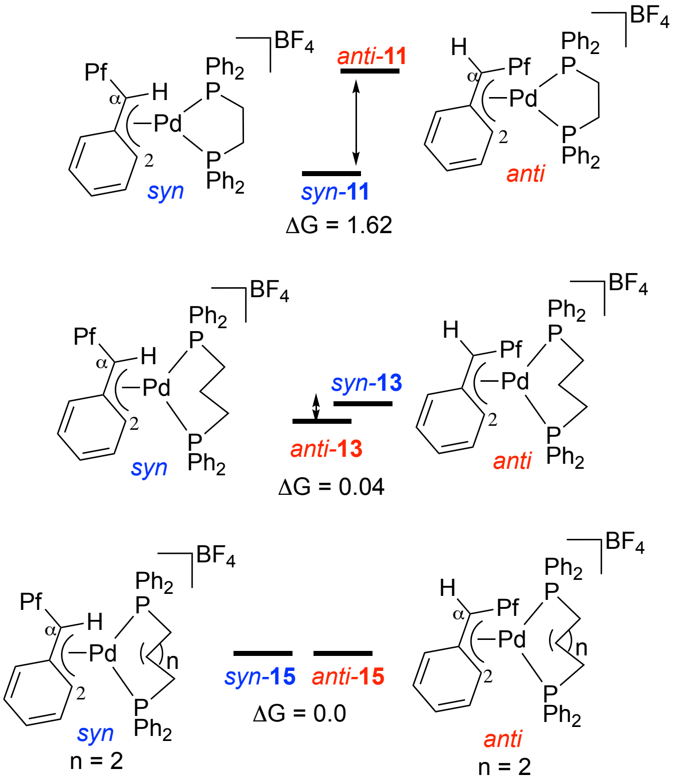

Figure S41. Gibbs energy difference in kcal mol^{-1} between the two isomers (*syn* or *anti*) in complexes **11**, **13** and **15**. (Pf = C_6F_5).

Table S4. Geometrical parameters extracted from DFT calculation of the optimized structure. Selected bond lengths (Å) and angles (º).

Geometrical parameters	*syn*-**11**	*anti*-**11**	*syn*-**13**	*anti*-**13**	*syn*-**15**	*anti*-**15**
P-Pd-P	85.4	85.3	96.3	93.2	98.8	98.0
Pd–C$^{\alpha}$	2.144	2.167	2.137	2.156	2.137	2.162
Pd-C^{2}	2.514	2.311	2.662	2.375	2.662	2.391
K_{eq}	6.5 10^{-2}		1.07		1.0	

Coordination angles in the palladium intermediates.

Table S5. Selected angles (º) from DFT calculations of the optimized structures.

	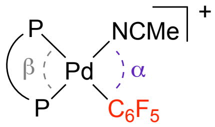		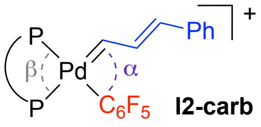	**I2-carb**	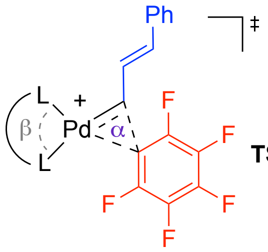 **TS-I2-allylPd**	
**P–P**	**β**	**α**	**β**	**α**	**β**	**α**
dppe	84.29	94.60	83.47	90.99	84.08	64.85
dppp	90.54	90.77	87.77	86.06	87.76	63.69
dppb	98.96	88.24	98.70	82.43	99.66	63.84

4.2. Gibbs energy profiles

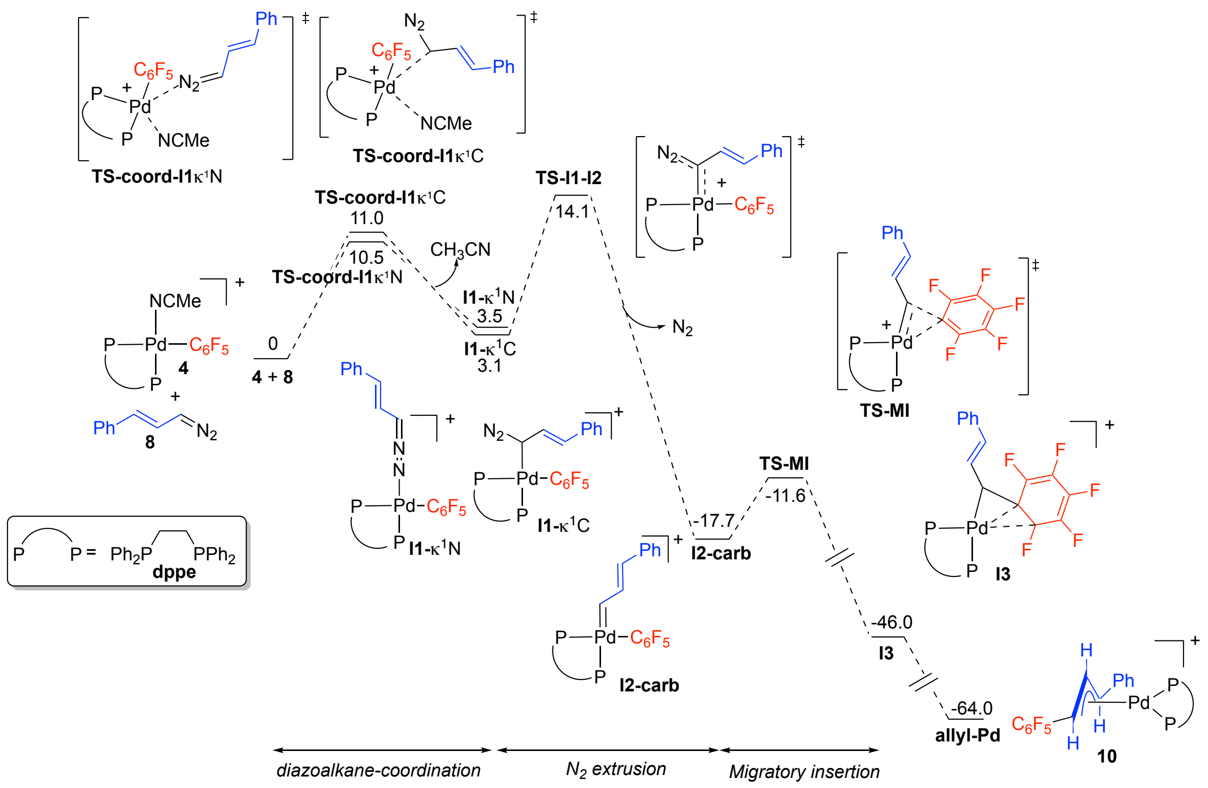

Figure S42. Gibbs energy profile of the reaction of the dppe palladium complex **4** with diazoalkane **8** to give the η^3-allylic complex **10** via carbene-aryl coupling. Energies in kcal mol^{-1}

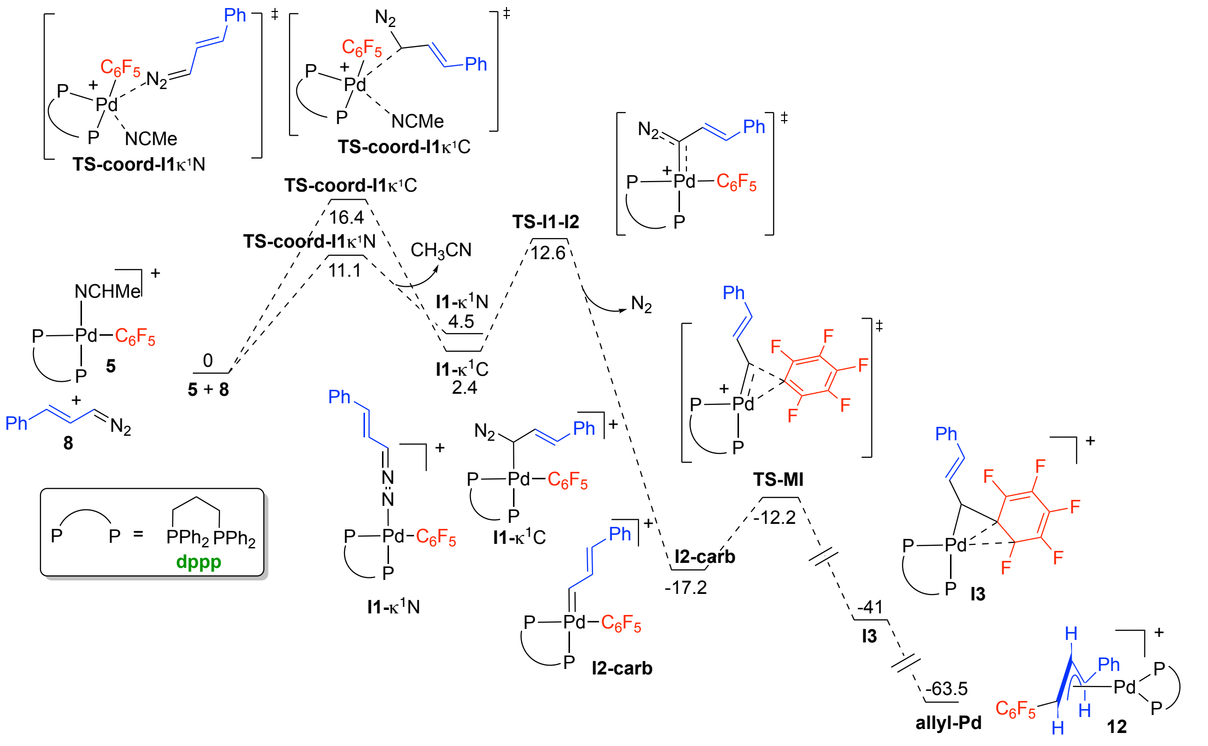

Figure S43. Gibbs energy profile of the reaction of the dppp palladium complex **5** with diazoalkane **8** to give the η^3-allylic complex **12** via carbene-aryl coupling. Energies in kcal mol^{-1}

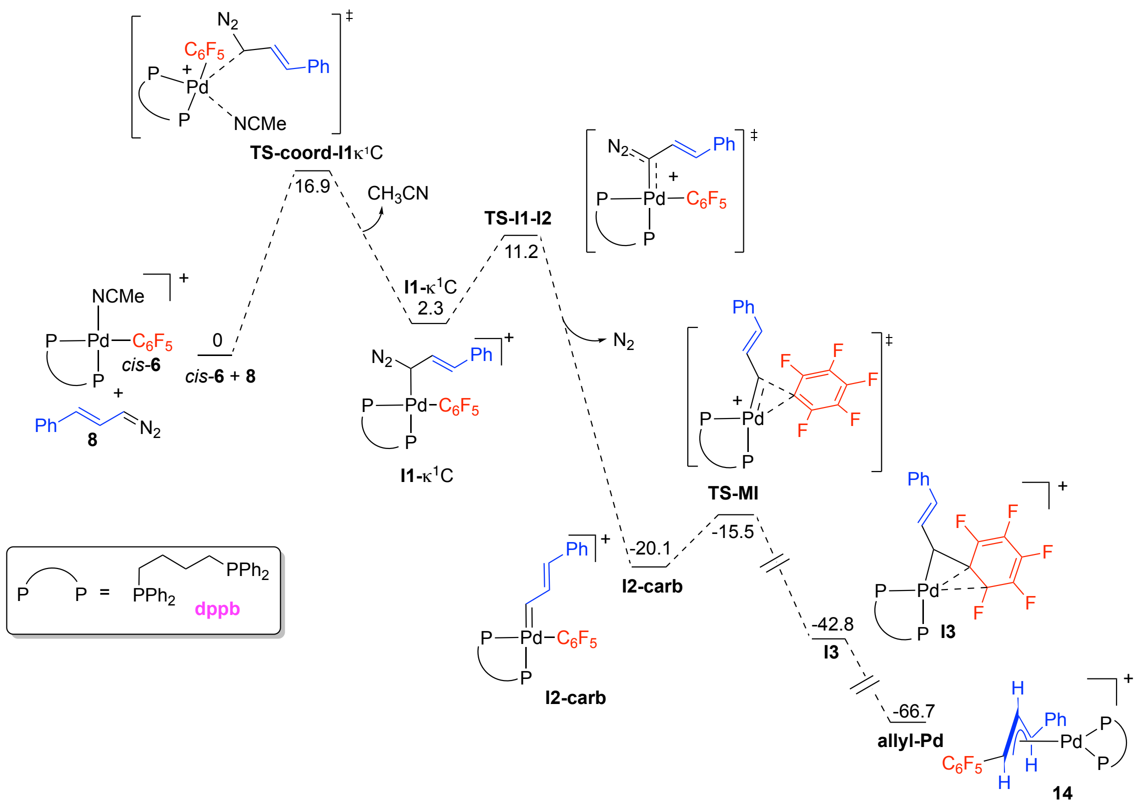

Figure S44. Gibbs energy profile of the reaction of the dppb palladium complex *cis*- **6** with diazoalkane **8** to give the η^3-allylic complex **14** via carbene-aryl coupling. Energies in kcal mol^{-1}

4.3. Probing a dissociative diazoalkane substitution for complex 4 (dppe).

A dissociative pathway, where the labile acetonitrile goes out of the coordination sphere affording a three-coordinated T-shaped intermediate **I4** can be proposed. The acetonitrile dissociation has a low energy cost (9.2 kcal mol^{-1}) to give the rather unstable intermediate **I4** (10.9 kcal mol^{-1}) as shown in Figure S45. We were unable to locate the transition state for the direct diazo coordination in a κ^1-C fashion to the three-coordinated intermediate **I4** due to the high stabilization afforded by the coordination of the double bond of the diazo compound (**I5**). The DFT energy profile scan in Figure S46 (upon variation of the C(diazoalkane)–Pd distance) shows that the diazoalkane **8** approaches the vacant coordination site in **I4** with a barrierless process, affording an intermediate with the olefin coordinated (**I5**). From this intermediate **I5** the rearrangement to reach a κ^1-C coordination mode of the diazoalkane is estimated in 11.2 kcal mol^{-1}, close to the energy barrier of the associative pathway.

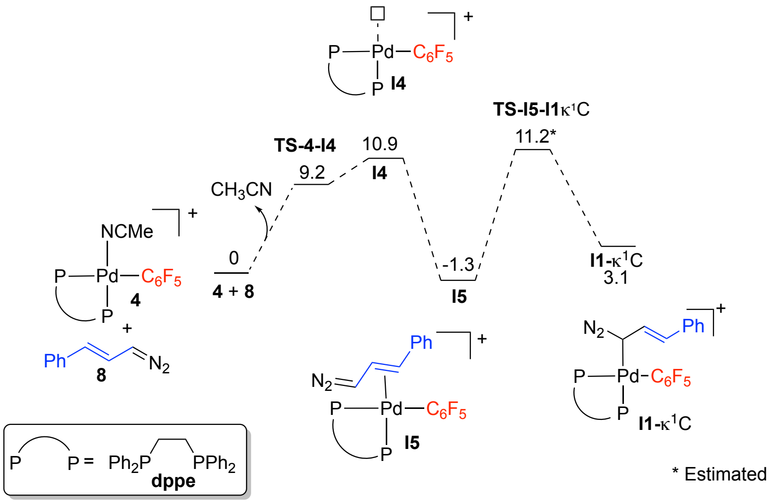

Figure S45. Gibbs energy profile of the coordination of the diazoalkane **8** to the palladium complex **4** via a dissociative pathway to give the intermediate **I1**-κ^1-C. **TS-I5- I1**-κ^1-C estimated according to Figure S46. Energies in kcal mol^{-1}

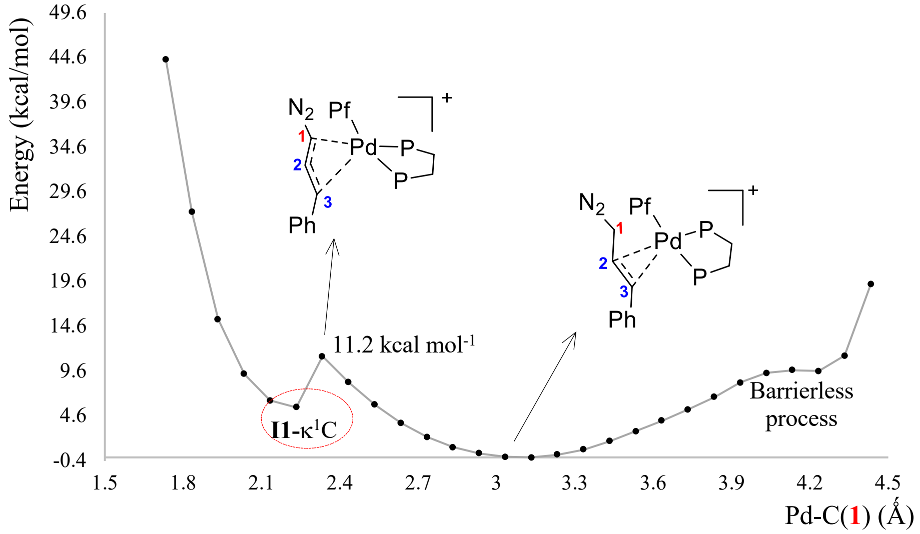

Figure S46. Energy scan for the coordination of diazo compound **8** in the κ^1-C mode to the three-coordinated intermediate **I4**. Pf = C_6F_5.

4.4. Calculated energies.

Calculated Potential Energies (atomic units).

SCF energy at high basis set level (Basis set II) and free energy correction at low basis set level (Basis set I). Then sum of both energies provides the final free energy of each compound used in the manuscript. Cartesian Coordinates (Å) can be found in a separate .xyz formatted document.

Some of the structures for the dppe intermediates and TSs (N_2 extrusion and migratory insertion) have been calculated and reported before,[4] but they are also included here for convenience and easy comparison.

NCMe
SCF Energy = -132.7043236000
Thermal Correction to Gibbs Free Energy = 0.021396

N_2
SCF Energy = -109.4851699000
Thermal Correction to Gibbs Free Energy = -0.012817

8 (diazoalkane)
SCF Energy = -456.9843711000
Thermal Correction to Gibbs Free Energy = 0.113084

***Dppe**-complexes*

4
SCF Energy = -2674.4945438000
Thermal Correction to Gibbs Free Energy = 0.447408

TS-coord-I1-κ^1C-dppe
SCF Energy = -3131.4845185000
Thermal Correction to Gibbs Free Energy = 0.5867470

I1-κ^1C-dppe
SCF Energy = -2998.7746861000
Thermal Correction to Gibbs Free Energy = 0.544117

TS-I1-I2-dppe
SCF Energy = -2998.7552974000
Thermal Correction to Gibbs Free Energy = 0.542265

I2-carb-dppe
SCF Energy = -2889.3048001000
Thermal Correction to Gibbs Free Energy = 0.535992

TS-MI-dppe
SCF Energy = -2889.2956795000
Thermal Correction to Gibbs Free Energy = 0.536691

I3-dppe
SCF Energy = -2889.3562344000
Thermal Correction to Gibbs Free Energy = 0.542396

10 (allylic Pd-complex)
SCF Energy = -2889.3843871000
Thermal Correction to Gibbs Free Energy = 0.541635

TS- coord-I1-κ^{1}N-dppe
SCF Energy = -3131.4830448000
Thermal Correction to Gibbs Free Energy = 0.5845130

I1-κ^{1}N-dppe
SCF Energy = -2998.7751202000
Thermal Correction to Gibbs Free Energy = 0.545220

***Dppp*-complexes**

5
SCF Energy = -2713.784106100
Thermal Correction to Gibbs Free Energy = 0.4761660

TS-coord-I1-κ^{1}C-dppp
SCF Energy = -3170.767933700
Thermal Correction to Gibbs Free Energy = 0.6177790

I1-κ^{1}C-dppp
SCF Energy = -3038.065787500
Thermal Correction to Gibbs Free Energy = 0.5732660

TS-I1-I2-dppp
SCF Energy = -3038.044076000
Thermal Correction to Gibbs Free Energy = 0.5678370

I2-carb-dppp
SCF Energy = -2928.593711700
Thermal Correction to Gibbs Free Energy = 0.5650000

TS-MI-dppp
SCF Energy = -2928.587108900
Thermal Correction to Gibbs Free Energy = 0.5663940

I3-dppp
SCF Energy = -2928.635727400
Thermal Correction to Gibbs Free Energy = 0.5688570

12 (allylic Pd-complex)
SCF Energy = -2928.674562000
Thermal Correction to Gibbs Free Energy = 0.571961

TS-coord-I1-κ^{1}N-dppp
SCF Energy = -3170.770384100
Thermal Correction to Gibbs Free Energy = 0.6118900

I1-κ^{1}N-dppp
SCF Energy = -3038.061352700
Thermal Correction to Gibbs Free Energy = 0.5721960

***Dppb**-complexes*

cis-**6**
SCF Energy = -2753.068677500
Thermal Correction to Gibbs Free Energy = 0.5044840

TS-coord-I1-κ^{1}C-dppb
SCF Energy = -3210.051655500
Thermal Correction to Gibbs Free Energy = 0.6460930

I1-κ^{1}C-dppb
SCF Energy = -3077.349495800
Thermal Correction to Gibbs Free Energy = 0.5994270

TS-I1-I2-dppb
SCF Energy = -3077.334366300
Thermal Correction to Gibbs Free Energy = 0.5997560

I2-carb-dppb
SCF Energy = -2967.884002100
Thermal Correction to Gibbs Free Energy = 0.5942940

TS-MI-dppb
SCF Energy = -2967.878137300
Thermal Correction to Gibbs Free Energy = 0.5958520

I3-dppb
SCF Energy = -2967.925868200
Thermal Correction to Gibbs Free Energy = 0.6000740

14 (allylic Pd-complex)
SCF Energy = -2967.962236200
Thermal Correction to Gibbs Free Energy = 0.5982980

***η^3*-benzylic** *complexes*

syn-**11**
SCF Energy = -2811.995810500
Thermal Correction to Gibbs Free Energy = 0.5086130

anti-**11**
SCF Energy = -2811.996575800
Thermal Correction to Gibbs Free Energy = 0.5119600

syn-**13**
SCF Energy = -2851.289908800
Thermal Correction to Gibbs Free Energy = 0.5351550

anti-**13**
SCF Energy = -2851.295325500
Thermal Correction to Gibbs Free Energy = 0.5405150

syn-**15**
SCF Energy = -2890.579877300
Thermal Correction to Gibbs Free Energy = 0.5631350

anti-**15**
SCF Energy = -2890.582694000
Thermal Correction to Gibbs Free Energy = 0.5675440

Dissociative pathway dppe-complexes

TS-4-I4
SCF Energy = -2674.4776082000
Thermal Correction to Gibbs Free Energy = 0.4451160

I4
SCF Energy = -2541.7552508000
Thermal Correction to Gibbs Free Energy = 0.4053710

I5
SCF Energy = -2998.7833619000
Thermal Correction to Gibbs Free Energy = 0.5458540

4.5. IRC for selected transition states.

Intrinsic reaction coordinate (IRC) calculations were carried out for the diazoalkane coodination and the N_2 extrusion transition states. The paths for dppb as bidentate phosphine are shown in Figures S47-S49. Gaussian16 was used as program package at the same level described in the computational details.[5] Forward and reverse paths were calculated separately.

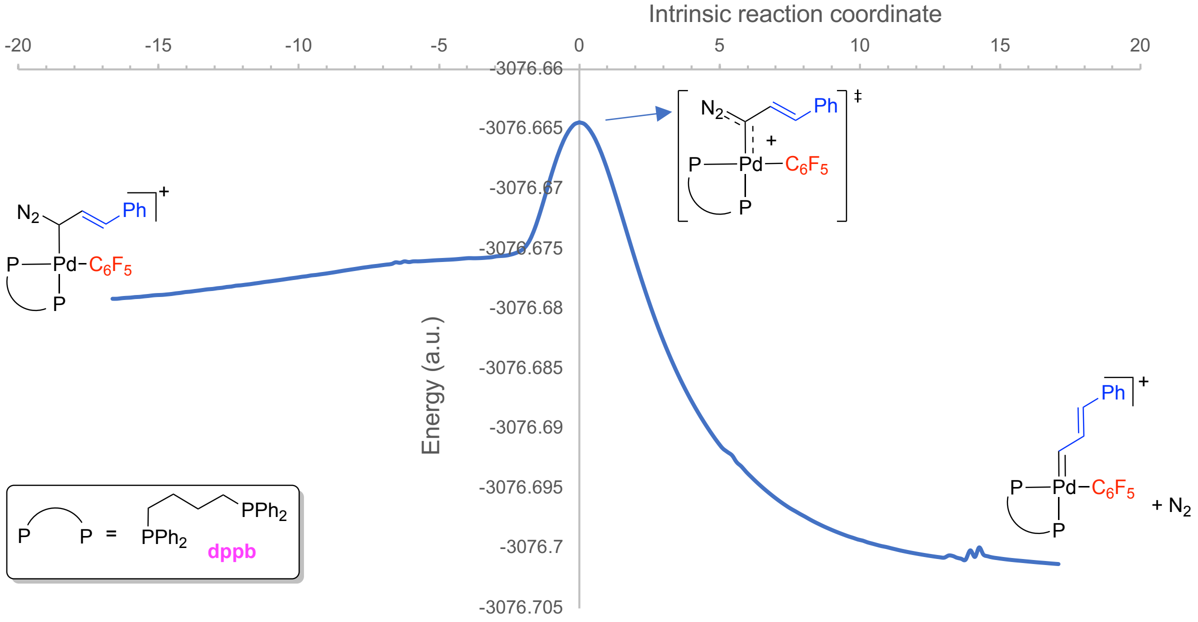

Figure S47. Intrinsic reaction coordinate (IRC) for the N_2 extrusion conecting the transition state **TS-I1-I2-dppb** to reactants (**I1-κ¹C-dppb**) and products (**I2-carb-dppb**).

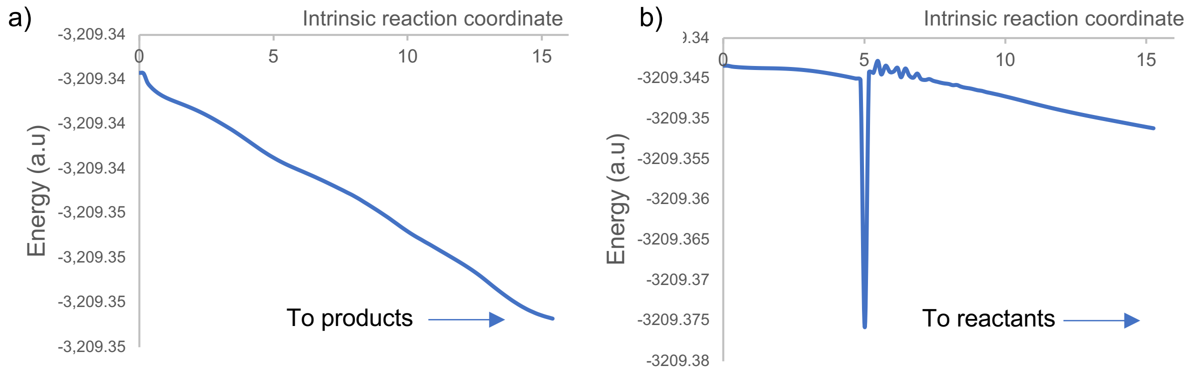

Figure S48. Intrinsic reaction coordinate (IRC) for the coordination of the diazoalkane to the dppb complex *cis*-**6**: a) Forward path (to products). b) Reverse path (to reactants); the low energy point in the latter corresponds to an intermediate test structure in the process where the bond lenghts of several atoms in the diphosphino backbone have been stretched. An inset of this path is used in Figure S49.

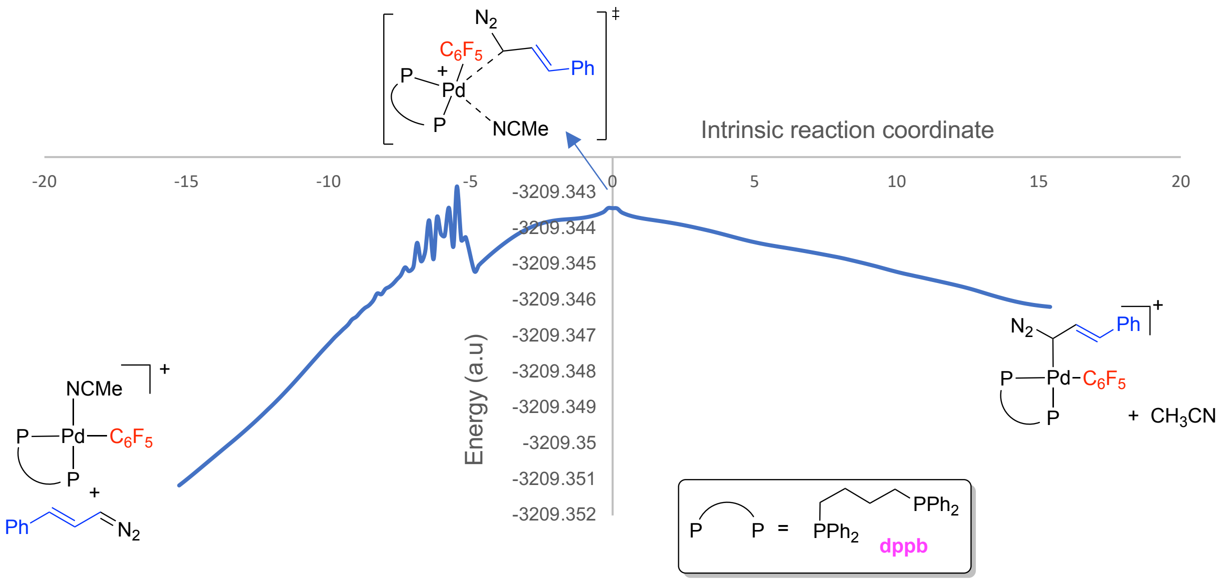

Figure S49. Intrinsic reaction coordinate (IRC) for the coordination of the diazoalkane to complex *cis*-**6** conecting the transition state **TS-coord-I1-κ^{1}C-dppb** to reactants (*cis*-**6**) and products (**I1-κ^{1}C-dppb**).

5. References

(1) CrysAlisPro Software system, version 1.171.33.51, 2009, Oxford Diffraction Ltd, Oxford, UK.

(2) Sheldrick, G. M. Crystal structure refinement with SHELXL. *Acta Cryst.*, **2015**, *C71*, 3-8.

(3) Dolomanov, O. V.; Bourhis, L. J.; Gildea, R. J.; Howard, J. A. K.; Puschmann, H. *OLEX2*: a complete structure solution, refinement and analysis program. *J. Appl. Crystallogr.* **2009**, *42*, 339-341.

(4) Villalba F.; Albéniz, A. C. Diazo compounds and palladium–aryl complexes: trapping the elusive carbene migratory insertion organometallic products. *Dalton Trans.* **2022***, 51,* 14847-14851.

(5) Gaussian 16, Revision C.01, Frisch, M. J.; Trucks, G. W.; Schlegel, H. B.; Scuseria, G. E.; Robb, M. A.; Cheeseman, J. R.; Scalmani, G.; Barone, V.; Petersson, G. A.; Nakatsuji, H.; Li, X.; Caricato, M.; Marenich, A. V.; Bloino, J.; Janesko, B. G.; Gomperts, R.; Mennucci, B.; Hratchian, H. P.; Ortiz, J. V.; Izmaylov, A. F.; Sonnenberg, J. L.; Williams-Young, D.; Ding, F.; Lipparini, F.; Egidi, F.; Goings, J.; Peng, B.; Petrone, A.; Henderson, T.; Ranasinghe, D.; Zakrzewski, V. G.; Gao, J.; Rega, N.; Zheng, G.; Liang, W.; Hada, M.; Ehara, M.; Toyota, K.; Fukuda, R.; Hasegawa, J.; Ishida, M.; Nakajima, T.; Honda, Y.; Kitao, O.; Nakai, H.; Vreven, T.; Throssell, K.; Montgomery, J. A., Jr.; Peralta, J. E.; Ogliaro, F.; Bearpark, M. J.; Heyd, J. J.; Brothers, E. N.; Kudin, K. N.; Staroverov, V. N.; Keith, T. A.; Kobayashi, R.; Normand, J.; Raghavachari, K.; Rendell, A. P.; Burant, J. C.; Iyengar, S. S.; Tomasi, J.; Cossi, M.; Millam, J. M.; Klene, M.; Adamo, C.; Cammi, R.; Ochterski, J. W.; Martin, R. L.; Morokuma, K.; Farkas, O.; Foresman, J. B.; Fox, D. J. Gaussian, Inc., Wallingford CT, 2016.
